# Green-Synthesized *Curcuma longa*-Derived Silver Nanoparticles for Oral Biomaterial Applications: Physicochemical Characterization, Antibacterial Activity, Preliminary Cytocompatibility and In Ovo Biocompatibility Screening

**DOI:** 10.3390/jfb17080357

**Published:** 2026-07-25

**Authors:** Mhd Kher Alsaeyd Ahmad, Doina Chioran, Dana-Emanuela Pitic (Coţ), Elena-Alina Moacă, Diana Haj Ali, Iasmina-Alexandra Predescu, Alina Hegheş, Cristina-Ioana Talpoş-Niculescu, Ramona-Amina Popovici, Ioana Macaşoi, Codruţa-Eliza Ille, Alfred Mark Sallai, Lucian Barbu-Tudoran, Mirela Voicu

**Affiliations:** 1 Doctoral School of Dental Medicine, “Victor Babeş” University of Medicine and Pharmacy, Revolutiei Ave. 1989, No. 9, 300580 Timisoara, Romania; mhd.alsaeyd-ahmad@umft.ro; 2 Faculty of Dental Medicine, “Victor Babeş” University of Medicine and Pharmacy, Revolutiei Ave. 1989, No. 9, 300580 Timisoara, Romania; chioran.doina@umft.ro (D.C.); dana.pitic@umft.ro (D.-E.P.); ramona.popovici@umft.ro (R.-A.P.); ille.codruta@umft.ro (C.-E.I.); sallai.mark@umft.ro (A.M.S.); 3 Faculty of Pharmacy, “Victor Babeş” University of Medicine and Pharmacy, 2 Eftimie Murgu Square, 300041 Timisoara, Romania; alina.moaca@umft.ro (E.-A.M.); diana.haj-ali@umft.ro (D.H.A.); iasmina-alexandra.predescu@umft.ro (I.-A.P.); macasoi.ioana@umft.ro (I.M.); voicu.mirela@umft.ro (M.V.); 4 Research Center for Pharmaco-Toxicological Evaluations, “Victor Babeş” University of Medicine and Pharmacy, 2 Eftimie Murgu Square, 300041 Timisoara, Romania; 5 Research Center of Digital and Advanced Technique for Endodontic, Restorative and Prosthetic Treatment (TADERP), “Victor Babeş” University of Medicine and Pharmacy, Revolutiei Ave. 1989, No. 9, 300580 Timisoara, Romania; 6 Faculty of Biology and Geology, “Babes-Bolyai” University, 5–7 Clinicilor Street, 400006 Cluj-Napoca, Romania; lucianbarbu@yahoo.com; 7 Electron Microscopy Integrated Laboratory, National Institute for R & D of Isotopic and Molecular Technologies, 67–103 Donat Street, 400293 Cluj-Napoca, Romania

**Keywords:** *Curcuma longa*, silver nanoparticles, green synthesis, antibacterial activity, minimum inhibitory concentration, human gingival fibroblasts, cytocompatibility, HET-CAM, oral biomaterials

## Abstract

Background/Objectives: Plant-mediated silver nanoparticles (AgNPs) are promising components for oral biomaterials because of their antimicrobial potential; however, their biological behavior depends strongly on the phytochemical matrix, physicochemical characteristics, and exposure concentration. This study aimed to evaluate silver nanoparticles formulations synthesized using turmeric powder-derived *Curcuma longa* ethanolic and aqueous extracts, with emphasis on physicochemical characterization, antibacterial activity against oral-relevant Gram-positive bacteria, cytocompatibility toward human gingival fibroblasts (HGF-1), and acute in ovo vascular compatibility. Methods: AgCUR-EtOH NPs and AgCUR-H_2_O NPs were synthesized using CUR-EtOH and CUR-H_2_O extracts as reducing and stabilizing matrices. The resulting formulations were characterized by UV–visible spectroscopy (UV-Vis), dynamic light scattering (DLS), zeta-potential analysis, X-ray diffraction (XRD), Fourier-transform infrared spectroscopy (FTIR), transmission electron microscopy (TEM), and energy-dispersive X-ray spectroscopy (EDX). Minimum inhibitory concentrations (MICs) and minimum bactericidal concentrations (MBCs) were determined against *Streptococcus mutans*, *Streptococcus oralis*, and *Staphylococcus aureus*. Cytocompatibility was evaluated in HGF-1 human gingival fibroblasts after 24 h exposure to 1–10 µg/mL using complementary viability, lysosomal, mitochondrial, and fluorescence-based assays. Acute vascular irritation was assessed using the hen’s egg test–chorioallantoic membrane (HET-CAM) assay. Results: Both formulations exhibited broad, polydisperse hydrodynamic distributions and negative apparent zeta potentials. AgCUR-H_2_O NPs showed a lower Z-average diameter than AgCUR-EtOH NPs under their respective solvent-specific measurement conditions. XRD pattern revealed heterogeneous crystalline compositions dominated by residual AgNO_3_, together with weaker contributions consistent with metallic Ag and a possible minor oxidized silver phase. FTIR spectra demonstrated extract-derived organic functional groups and prominent nitrate-associated bands. TEM/EDX confirmed Ag-containing nanostructures with approximate size ranges of 15–175 nm for AgCUR-EtOH NPs and 15–150 nm for AgCUR-H_2_O NPs. *S. mutans* was the most susceptible microorganism, with MIC values of 9 and 7 µg/mL and MBC values of 88 and 62 µg/mL for AgCUR-EtOH NPs and AgCUR-H_2_O NPs, respectively. AgCUR-H_2_O NPs consistently showed lower MIC and MBC values against all tested strains, but also produced a more pronounced concentration-dependent reduction in HGF-1 viability. At 10 µg/mL, cell viability was 71.88% for AgCUR-EtOH NPs and 52.14% for AgCUR-H_2_O NPs. Both formulations showed low acute irritation potential in ovo, with irritation scores of 1.06 and 0.69, respectively. Conclusions: The two CUR-AgNP formulations exhibited distinct physicochemical, antibacterial, and cellular response profiles under the tested conditions. At equivalent concentrations expressed as total dried formulation mass, AgCUR-H_2_O NPs yielded lower MIC and MBC values against the tested bacterial strains, whereas AgCUR-EtOH NPs produced a less pronounced reduction in HGF-1 viability. Because the powders were not quantitatively normalized for total silver, extract-derived organic fraction, or residual precursor content, these differences cannot be attributed exclusively to nanoparticle properties or to the extraction solvent and should not be interpreted as evidence of the intrinsic superiority of either formulation. Both formulations showed low acute vascular irritation. Further quantitative compositional, silver-release, and biofilm assessments are required before incorporation into oral biomaterial platforms.

## 1. Introduction

Nanotechnology has become an important direction in biomedical and dental materials research, allowing the development of nanoscale systems with improved surface reactivity, antimicrobial performance, and biological functionality. Among metallic nanoparticles, silver nanoparticles (AgNPs) have attracted particular attention due to their broad-spectrum antimicrobial activity and their potential incorporation into wound dressings, restorative materials, endodontic formulations, prosthetic devices, implant coatings, and other antimicrobial biomaterials [1,2,3,4]. Their antimicrobial effects are generally associated with multiple mechanisms, including silver ion release, reactive oxygen species generation, bacterial membrane disruption, interaction with thiol-containing proteins, and interference with DNA replication, which together contribute to reduced microbial viability and biofilm formation [2,4,5,6]. In dentistry, AgNPs have therefore been investigated as functional additives for infection control in restorative, prosthetic, implant-related, and endodontic applications [7,8,9]. However, their biological response is strongly influenced by particle size, concentration, surface chemistry, stabilizing agents, exposure time, and the target cell type. Consequently, the antimicrobial potential of AgNPs must be balanced by careful cytocompatibility and biocompatibility assessment before considering their use in oral-health-oriented formulations [1,2,3].

Although conventional physical and chemical methods can produce AgNPs with controlled size and morphology, they often require strong reducing agents, synthetic stabilizers, organic solvents, or energy-demanding conditions, which may limit their suitability for biomedical-oriented applications. In this context, green synthesis has emerged as a more sustainable alternative, aiming to reduce the use of hazardous reagents while maintaining the functional properties of AgNPs [10,11]. Among green approaches, plant extract-mediated synthesis is particularly attractive because phytochemicals naturally present in the extracts can act simultaneously as reducing agents for Ag^+^ conversion to metallic Ag^0^ and as capping/stabilizing agents for the newly formed nanoparticles [11,12]. Polyphenols, flavonoids, terpenoids, proteins, sugars, and other plant-derived metabolites may participate in silver ion reduction, surface stabilization, and modulation of nanoparticle growth. Consequently, the plant species, plant part, extraction solvent, phytochemical composition, pH, temperature, reaction time, and silver precursor concentration can influence nanoparticle size, morphology, stability, and biological activity [12,13,14]. Therefore, plant-mediated AgNP synthesis is not only an eco-friendlier production strategy, but also a formulation-dependent process in which the extract composition may strongly affect the physicochemical and biological profile of the resulting nanoparticles.

*Curcuma longa* L., commonly known as turmeric, represents a valuable phytochemical source for plant-mediated nanoparticle synthesis due to its complex composition, which includes curcuminoids, phenolic acids, flavonoids, terpenoids, volatile oils, and other secondary metabolites with antioxidant and redox-active properties. These phytochemicals may participate in the reduction of Ag^+^ to Ag^0^ and may also contribute to nanoparticle capping and stabilization. Previous studies have shown that turmeric-derived extracts can mediate the green synthesis of AgNPs, with nanoparticle formation commonly confirmed by UV–Vis spectroscopy and electron microscopy-based techniques [15,16,17]. For example, Shameli et al. reported the biosynthesis of AgNPs using *Curcuma longa* tuber-powder extract, which acted simultaneously as a reducing and stabilizing agent, while Alsammarraie et al. used aqueous turmeric powder extract for AgNP synthesis and antimicrobial evaluation. Moreover, metabolomic and phytochemical studies support the chemical complexity of *Curcuma longa*, emphasizing that extract composition depends strongly on the extraction procedure and solvent [18,19]. In this regard, a recent study from our group showed that the turmeric powder-derived CUR-EtOH and CUR-H_2_O extracts differed in extraction yield, antioxidant potential, total phenolic content, targeted polyphenolic profile, and preliminary biological behavior, supporting their consideration as chemically and biologically distinct extract matrices [20]. Therefore, aqueous and ethanolic turmeric extracts should be regarded as distinct phytochemical matrices, potentially leading to differences in AgNP formation, optical behavior, dispersion characteristics, crystalline-phase composition, surface-associated chemistry, and biological response.

Despite their antimicrobial potential, AgNPs may exert concentration-, size-, and surface chemistry-dependent effects on mammalian cells; therefore, as for other dental biomaterials, preliminary biological evaluation is essential before considering their clinical or regenerative use, since material composition and surface-related properties may influence local cellular responses and tissue compatibility [21,22,23]. Human gingival fibroblasts are particularly relevant for this type of evaluation because they represent a major cellular component of the gingival connective tissue and are directly involved in extracellular matrix homeostasis, wound healing, and local inflammatory responses. Previous studies using HGF-1 or related oral fibroblast models have shown that the biological response to AgNPs depends not only on silver concentration but also on particle size, intracellular silver accumulation, and surface functionalization. For example, Niska et al. demonstrated that AgNP cytotoxicity toward HGF-1 cells was strongly influenced by the capping agent, with lipoic acid- and polyethylene glycol-capped nanoparticles showing better cytocompatibility than uncapped or tannic-acid-capped AgNPs [24]. Similarly, Zorraquín-Peña et al. reported concentration-dependent cytotoxicity and inflammatory responses in oral cells exposed to glutathione-stabilized AgNPs [25]. These findings support the need for a formulation-specific biological evaluation of newly synthesized AgNPs. Moreover, because metabolic activity alone does not fully describe cell health, multiparametric screening approaches combining viability, morphology, lysosomal integrity, mitochondrial function, nuclear alterations, and membrane integrity provide a more reliable preliminary assessment of nanoparticle cytocompatibility.

Although in vitro assays provide essential information on cell-specific responses, they do not reproduce the complexity of a vascularized biological membrane. Therefore, complementary in ovo models can strengthen preliminary biocompatibility screening by providing additional information on acute vascular irritation. The hen’s egg test–chorioallantoic membrane (HET-CAM) assay is a widely used alternative model for evaluating irritation potential, allowing the direct observation of early vascular events such as hemorrhage, vascular lysis, and coagulation after topical application of a test substance [26,27]. In the field of nanomaterial safety assessment, the chorioallantoic membrane model has also been proposed as a useful intermediate platform between conventional cell culture assays and more complex in vivo studies, particularly because it enables rapid visualization of vascular responses to nanoscale materials [28]. Previous work evaluating AgNP colloids using the hen’s egg test has shown that CAM-based screening can be combined with antimicrobial assessment relevant to oral applications [29]. However, limited information is available regarding the side-by-side comparison of AgNPs synthesized from aqueous and ethanolic turmeric-derived extracts within a single experimental framework integrating detailed physicochemical characterization, antibacterial assessment against oral-relevant microorganisms, cytocompatibility in HGF-1 gingival fibroblasts, and acute vascular irritation using the HET-CAM model. Such an approach may help clarify how solvent-dependent phytochemical matrices influence nanoparticle formation and the balance between antibacterial efficacy and biological compatibility.

In oral tissue regeneration, smart biomaterials are increasingly designed to combine structural support with bioactive, antimicrobial, and immunomodulatory functions [30,31]. In periodontal and mucosal wound-healing applications, infection control and fibroblast compatibility are particularly important, as excessive microbial colonization and impaired soft-tissue cell responses may compromise healing and integration. In this context, plant-mediated AgNPs may act as multifunctional nanocomponents for regenerative platforms, provided that their antimicrobial potential is balanced by acceptable cytocompatibility toward oral cells [30,32].

Previous studies have demonstrated the feasibility of *Curcuma longa*-turmeric-mediated AgNP synthesis, generally emphasizing nanoparticle formation, selected physicochemical characteristics, and antimicrobial applications. For example, Shameli et al. reported the biosynthesis of AgNPs using *Curcuma longa* tuber-powder extract as both a reducing and stabilizing agent, whereas Alsammarraie et al. used aqueous turmeric-powder extract for AgNP synthesis and evaluated antibacterial activity against food-borne pathogens. Maghimaa and Alharbi further extended this approach toward antimicrobial textile coatings and wound-healing-related applications using *Curcuma longa*-derived AgNPs [15,16,17]. However, integrated comparisons of AgNP formulations obtained from solvent-dependent turmeric extract matrices, particularly within an oral-biomaterial-oriented experimental framework, remain limited. The novelty of the present study lies in the side-by-side evaluation of AgNPs synthesized using turmeric powder-derived ethanolic and aqueous extracts previously shown to differ in their phytochemical and biological profiles [20]. The study combines complementary physicochemical characterization with MIC and MBC determination against oral-relevant Gram-positive bacteria, multiparametric cytocompatibility assessment in HGF-1 human gingival fibroblasts, and acute vascular irritation screening using the HET-CAM model. This integrated design links the extraction solvent and formulation characteristics to antibacterial activity and biological compatibility relevant to future oral biomaterial development.

In the context of the above, the aim of this study was to biosynthesize and evaluate silver nanoparticle formulations using turmeric powder-derived *Curcuma longa* ethanolic and aqueous extracts as distinct reducing and stabilizing matrices. The specific objectives were to: (i) establish the formulation-dependent physicochemical profiles of AgCUR-EtOH NPs and AgCUR-H_2_O NPs using UV–Vis spectroscopy, DLS, zeta-potential analysis, XRD, FTIR spectroscopy, TEM, and EDX; (ii) determine their MIC and MBC values against *Streptococcus mutans*, *Streptococcus oralis*, and *Staphylococcus aureus*; (iii) evaluate their concentration-dependent cytocompatibility in HGF-1 human gingival fibroblasts using complementary metabolic, lysosomal, mitochondrial, morphological, and fluorescence-based endpoints; and (iv) assess their acute vascular irritation potential using the HET-CAM assay. Through this integrated approach, the study sought to determine how the extraction solvent and resulting formulation characteristics influence the balance between antibacterial activity and biological compatibility, with relevance to the future development of oral biomaterial platforms.

## 2. Materials and Methods

### 2.1. Biosynthesis and Characterization of Turmeric-AgNPs

Two lyophilized extracts, in 96% ethanol and distilled water, were prepared from turmeric powder (*Curcuma longa*) to develop silver nanoparticles (AgNPs). The protocol for turmeric powder-derived *Curcuma longa* ethanolic and aqueous extracts (CUR-EtOH and CUR-H_2_O), as well as their characterization, is presented in detail in a recent publication of our research group [20]. The protocol for the biosynthesis of AgNPs was similar to the one described by Sarău et al. [33], with slight modifications, and consisted of: 500 mg of each lyophilized extract was resuspended in the corresponding solvent (96% ethanol and distilled water) until a final concentration of 10 mg/mL was obtained. Furthermore, 50 mL of each extract was subjected to a Heidolph MR Hei-Tec magnetic stirrer (Heidolph Instruments GmbH & Co. KG, Schwabach, Germany) at 250 rpm. When both extracts reached 60 °C, a freshly prepared aqueous solution of 1 M AgNO_3_ was added at an extract-to-AgNO_3_ volume ratio of 2:1. The ethanolic-extract-mediated reaction was allowed to proceed for a total of 4 h. In contrast, the aqueous-extract-mediated reaction was conducted for 3 h. The resulting formulations were denoted AgCUR-EtOH NPs and AgCUR-H_2_O NPs, respectively. After the green-synthesized CUR-AgNPs were obtained, they were centrifuged at 6000 rpm for 30 min and dried at 40 °C in the oven (POL-EKO Aparatura, Wodzisław Slaski, Poland) until completely dry. After that, each sample was hand-ground until a fine powder was obtained.

The reduction of silver ions by turmeric powder-derived *Curcuma longa* ethanolic and aqueous extracts during the synthesis of AgCUR-EtOH NPs and AgCUR-H_2_O NPs was monitored by UV–Vis spectroscopy using a UviLine 9400 spectrophotometer from SI Analytics (Mainz, Germany). Aliquots were withdrawn from the ongoing reactions at selected time points. For the comparative time-course presentation, spectra recorded after 15 min, 30 min, and 1, 2, and 3 h were considered for both reaction systems, providing a common monitoring interval. The ethanolic-extract-mediated reaction was subsequently continued for a total of 4 h before nanoparticle recovery, whereas the aqueous-extract-mediated reaction was completed after 3 h. AgCUR-EtOH reaction aliquots were diluted at a ratio of 1:1 using ethanol, with ethanol used as the instrumental blank. AgCUR-H_2_O reaction aliquots were diluted at a ratio of 1:20 using distilled water, with distilled water used as the corresponding blank. Spectra were recorded at room temperature in quartz cuvettes with an optical path length of 10 mm over the wavelength range of 190–1100 nm, at a spectral resolution of 2 nm. Because matrix-specific dilution conditions and different diluents were required to obtain interpretable spectral profiles, the raw absorbance intensities of the two formulations were not used for direct quantitative comparison. For comparison of the final visible-region spectral profiles, the spectra recorded after 3 h were normalized to their respective absorbance at 400 nm according to the following equation:

(1) $$A_{rel}\left(\lambda \right)=\;\frac{A_{\lambda }}{A_{400}}$$

where $A_{rel}(\lambda )$ is the relative absorbance at wavelength *λ*, $A_{\lambda }$ is the measured absorbance at the corresponding wavelength, and $A_{400}$ is the absorbance measured at 400 nm for the same spectrum. Normalized spectra were interpreted only in terms of relative spectral shape and slope and not as quantitative indicators of nanoparticle concentration or synthesis yield. UV–Vis measurements represented sequential aliquots collected from one synthesis batch per formulation and were therefore interpreted descriptively.

The Z-average hydrodynamic diameter and polydispersity index (PDI) of redispersed AgCUR-EtOH NPs and AgCUR-H_2_O NPs were determined by dynamic light scattering (DLS) using a Zetasizer Nano ZS instrument (Malvern Instruments, Worcestershire, UK). For each formulation, 50 mg of dried nanoparticle powder was dispersed in 20 mL of the corresponding dispersant, resulting in a final concentration of 2.5 mg/mL. AgCUR-H_2_O NPs were redispersed in distilled water, whereas AgCUR-EtOH NPs were redispersed in ethanol, corresponding to the solvent system used for the respective extract-mediated formulation. Before analysis, the dispersions were sonicated for 10 min using a Q700 Sonicator (Qsonica, Newtown, CT, USA), operated in pulsed mode at 20% amplitude using a 5 s on/2 s off cycle. Measurements were performed at 30 °C, and intensity- and number-weighted hydrodynamic size distributions were generated using the instrument software.

The apparent zeta potential was determined by electrophoretic light scattering using the electrophoretic measurement cell of the same instrument. Five consecutive determinations were performed for each formulation in its corresponding dispersant, and the results were expressed as mean values ± standard deviation in millivolts. The obtained values were used to characterize the apparent surface charge and electrokinetic behavior of the redispersed formulations under the tested solvent-specific conditions.

The crystal structure and phase composition of the dried AgCUR-EtOH NPs and AgCUR-H_2_O NPs formulations were examined by powder X-ray diffraction using a Rigaku Ultima IV diffractometer (Rigaku, Tokyo, Japan). Diffraction measurements were carried out at room temperature (24 °C) using monochromated Cu Kα radiation with a wavelength of 0.15406 nm. The X-ray tube was operated at 40 kV and 40 mA, and the diffraction profiles were collected over a 2θ range of 10–80°.

Crystalline phase identification was performed by comparing the experimental diffraction profiles with reference patterns for AgNO_3_ (COD 1509468), face-centered cubic metallic Ag (COD 9008459), and Ag_6_O_2_ (COD 7109278) obtained from the Crystallography Open Database [34]. For graphical presentation, the experimental profiles were baseline-shifted, normalized independently to their respective maximum intensity, and vertically offset to facilitate visual comparison. No smoothing or interpolation was applied to the experimental diffraction data.

The apparent coherent crystallite size of the residual crystalline AgNO_3_ phase was estimated from four comparatively intense and resolved reflections at approximately 2θ = 19.6°, 21.7°, 24.3°, and 29.6°, using the Debye–Scherrer equation [35,36]:

(2) $$D_{XRD}=\;\frac{K\times \lambda }{\beta \times \cos\theta }$$

where *D_XRD_* represents the apparent crystallite size, *K* is the shape factor (0.9), *λ* is the Cu Kα wavelength (0.15406 nm), *β* is the FWHM of the selected reflection expressed in radians, and *θ* is the Bragg angle.

Peak positions and FWHM values were obtained by nonlinear least-squares fitting of the raw experimental diffraction profiles using a pseudo-Voigt peak function and a linear background. Peak fitting and numerical calculations were performed using Python 3.13 and SciPy version 1.17.0. The four individual crystallite-size estimates were used to calculate the arithmetic mean and standard deviation for each formulation. The results were expressed as the arithmetic mean ± standard deviation calculated across the four selected diffraction reflections. No correction for instrumental broadening was applied. The calculation was restricted to the residual AgNO_3_ phase because the weak metallic Ag reflections overlapped with those of AgNO_3_ and Ag_6_O_2_.

The functional groups present in the dried AgCUR-EtOH NPs and AgCUR-H_2_O NPs formulations were investigated by Fourier-transform infrared spectroscopy (FTIR) using a Prestige-21 FTIR spectrometer (Shimadzu, Duisburg, Germany). The dried powders were individually mixed with KBr and compressed into pellets. Spectra were recorded at room temperature in transmittance mode over the 4000–400 cm^−1^ spectral range, using a resolution of 4 cm^−1^.

Spectral interpretation was based on the position and profile of the main absorption bands and on comparison with published vibrational assignments for *Curcuma longa*-derived phytochemicals, curcumin-containing silver formulations, nitrate species, and silver-containing materials. Because the measurements were performed using separately prepared KBr pellets, absolute transmittance intensities were not used for direct quantitative comparison between the two formulations.

Transmission electron microscopy (TEM) coupled with energy-dispersive X-ray spectroscopy (TEM/EDX) was used to evaluate the morphology, size range, and elemental composition of the green-synthesized CUR-AgNPs. For TEM analysis, the dried AgCUR-EtOH NPs and AgCUR-H_2_O NPs powders were redispersed in distilled water and sonicated for several minutes to improve particle dispersion. Then, 7 μL of each nanoparticle suspension was deposited onto carbon-coated copper grids and allowed to dry at room temperature (23 ± 1 °C). Subsequently, the grids containing the dried nanoparticle samples were sputter-coated with a 6 nm carbon layer (Agar Automatic Sputter Coater, Essex, UK) to improve conductivity and imaging quality. The samples were examined using a Hitachi HD2700 cold field emission gun STEM microscope (Chiyoda, Tokyo, Japan), equipped with two windowless X-Max^N^ 100 EDX detectors from Oxford Instruments (Abingdon, UK). The images were acquired in bright-field STEM mode at an accelerating voltage of 30 kV. The approximate particle size range was estimated from calibrated representative TEM/STEM micrographs based on measurements performed using the microscope acquisition software. EDX analysis was performed to verify the presence of elemental silver in the analyzed samples.

### 2.2. Antibacterial Activity of CUR-AgNPs

The antibacterial activity of AgCUR-EtOH NPs and AgCUR-H_2_O NPs was evaluated by determining the minimum inhibitory concentration (MIC) and minimum bactericidal concentration (MBC), following the broth microdilution procedure described by Sarău et al. [33], in accordance with internationally accepted dilution susceptibility-testing principles [37,38]. The antibacterial assessment was performed against three Gram-positive bacterial strains: *Streptococcus mutans* ATCC 25175, *Streptococcus oralis* ATCC 9811, and *Staphylococcus aureus* ATCC 25923. The bacterial strains were obtained from the American Type Culture Collection (ATCC, Manassas, VA, USA). Fresh bacterial cultures were used to prepare suspensions adjusted to a turbidity equivalent to the 0.5 McFarland standard. The standardized inocula were subsequently diluted in sterile 0.85% NaCl solution to obtain a final bacterial density of approximately 5 × 10^5^ colony-forming units per milliliter.

Stock dispersions of AgCUR-EtOH NPs and AgCUR-H_2_O NPs were freshly prepared in ultrapure deionized water at a concentration of 1 mg/mL, without a prior sonication step. Appropriate working concentrations were prepared from the stock dispersions. The bacterial suspensions and the tested CUR-AgNP formulations were added to Mueller–Hinton broth and incubated for 24 h at 35 °C.

The MIC was defined as the lowest concentration of the tested formulation that prevented visible bacterial growth after incubation. To determine the MBC, 1 µL aliquots were subcultured from assay conditions showing no visible growth onto Columbia agar supplemented with 5% sheep blood. The MBC was defined as the lowest concentration resulting in a reduction of at least 99.9% of the initial bacterial population. All determinations were performed in triplicate for each bacterial strain and nanoparticle formulation. MIC and MBC values were expressed in µg/mL of total dried AgCUR-EtOH NPs or AgCUR-H_2_O NPs formulation present in the final assay medium. Therefore, the reported concentrations refer to the total formulation mass and not to elemental silver, metallic AgNP content, or AgNO_3_ concentration.

### 2.3. Preliminary In Vitro Cytocompatibility Screening of CUR-AgNPs

#### 2.3.1. Reagents and Equipment

Dulbecco’s Modified Eagle’s Medium (DMEM), fetal bovine serum (FBS), Penicillin/Streptomycin, the MTT assay kit, Acridine Orange, and Propidium Iodide were purchased from Sigma-Aldrich (Merck KGaA, Darmstadt, Germany). The JC-1 mitochondrial membrane potential assay kit was obtained from Elabscience (Houston, TX, USA), while trypsin-EDTA solution and phosphate-buffered saline (PBS) were purchased from ATCC (Manassas, VA, USA), and dimethyl sulfoxide (DMSO) was supplied by PanBiotech (Aidenbach, Germany). Ultrapure distilled water, Hoechst 33342, and MitoTracker^TM^ Red CMXRos were purchased from Thermo Fisher Scientific (Waltham, MA, USA).

In vitro analyses were performed using the Lionheart^TM^ FX automated microscope, the Cytation^TM^ 5 multimode microplate reader, and Gen5^TM^ Microplate Data Collection and Analysis Software (version 3.14) (BioTek Instruments Inc., Winooski, VT, USA).

#### 2.3.2. Cell Line and Culture Conditions

The HGF-1 human gingival fibroblast cell line (CRL-2014) was obtained from American Type Culture Collection (ATCC, Manassas, VA, USA). Cells were maintained under standard conditions (37 °C, 5% CO_2_) in Dulbecco’s Modified Eagle’s Medium (DMEM) supplemented with 10% fetal bovine serum (FBS) and 1% penicillin/streptomycin. Stock solutions of AgCUR-EtOH nanoparticles (AgCUR-EtOH NPs) and AgCUR-H_2_O nanoparticles (AgCUR-H_2_O NPs) were prepared at a concentration of 2 mg/mL in ultrapure distilled water. Working solutions were subsequently obtained by dilution in the culture medium to final concentrations of 1, 2, 3, 4, 5, and 10 μg/mL. The concentration range of 1–10 µg/mL was selected to characterize the lower-dose response of HGF-1 gingival fibroblasts, identify potentially better-tolerated exposure levels for the two CUR-AgNP formulations, and account for previously reported biological effects of CUR and AgNPs [39,40,41].

#### 2.3.3. Cell Viability Using the MTT Assay

Cell viability was evaluated using the MTT assay. HGF-1 cells were seeded in 96-well plates at a density of 1 × 10^4^ cells/well and allowed to adhere until the desired confluence was reached. Subsequently, the cells were exposed to increasing concentrations of AgCUR-EtOH NPs and AgCUR-H_2_O NPs for 24 h at 1–10 μg/mL. Following the treatment period, the medium was carefully replaced with fresh medium, and 10 μL/well of MTT reagent was added. After a 3 h incubation period, 100 μL/well of solubilization solution was added, and the plates were maintained at room temperature in the dark for 30 min. The absorbance was then recorded at 570 nm and 630 nm using a Cytation^TM^ 5 microplate reader. Cell viability was calculated after background correction by subtracting the absorbance at 630 nm from the absorbance at 570 nm and was expressed as a percentage relative to the untreated control group.

#### 2.3.4. Cell Morphology Assessment Using Bright-Field Microscopy

Morphological changes in HGF-1 cells were evaluated after 24 h of exposure to AgCUR-EtOH NPs and AgCUR-H_2_O NPs at concentrations ranging from 1 to 10 μg/mL. After treatment, representative images of treated and untreated control cells were acquired under bright-field illumination using a Lionheart^TM^ FX automated microscope. The images were processed and analyzed using Gen5^TM^ Microplate Data Collection and Analysis Software (version 3.14; BioTek Instruments Inc., Winooski, VT, USA).

#### 2.3.5. Neutral Red Uptake Assay

The neutral red uptake (NRU) assay was used to evaluate lysosomal integrity in HGF-1 cells after exposure to AgCUR-EtOH NPs and AgCUR-H_2_O NPs. Briefly, HGF-1 cells were seeded in 96-well flat-bottom plates at a density of 1 × 10^4^ cells/well and allowed to adhere. The cells were then exposed for 24 h to AgCUR-EtOH NPs and AgCUR-H_2_O NPs at concentrations ranging from 1 to 10 μg/mL. Following treatment, the culture medium was replaced with 100 μL/well of neutral red solution (40 μg/mL in culture medium), and the plates were incubated for 2 h at 37 °C in a 5% CO_2_ atmosphere to allow intracellular dye accumulation. Subsequently, the cells were gently washed with 150 μL PBS/well and imaged under bright-field conditions using a Lionheart^TM^ FX automated microscope. For dye extraction, 150 μL/well of destaining solution, consisting of approximately 50% ethanol, 49% ultrapure water, and 1% glacial acetic acid, was added. The absorbance of the released dye was measured at 540 nm using a Cytation^TM^ 5 plate reader. NRU values were expressed as percentages relative to the untreated control group. The procedure was performed in accordance with the method described by Vicaș et al. [42].

#### 2.3.6. Mitochondrial Membrane Potential (ΔΨm) Assay Using JC-1 Staining

Changes in mitochondrial membrane potential (ΔΨm) were evaluated in HGF-1 cells using the JC-1 fluorescence assay, according to the manufacturer’s instructions. Briefly, cells were seeded in black-walled, clear-bottom 96-well plates at a density of 1 × 10^4^ cells/well and allowed to reach approximately 70% confluence. The cells were then exposed for 24 h to AgCUR-EtOH NPs and AgCUR-H_2_O NPs at concentrations ranging from 1 to 10 μg/mL. After treatment, the culture medium was removed, and the cells were carefully washed with PBS. Subsequently, the cells were incubated with JC-1 staining solution (5 μM, 100 μL/well) for 45 min at 37 °C. After incubation, excess dye was removed by washing the cells twice with PBS. Representative fluorescence images were acquired using a Lionheart^TM^ FX automated microscope, and image analysis was performed using Gen5^TM^ Microplate Data Collection and Analysis Software. Fluorescence signals corresponding to JC-1 aggregates and monomers were measured at 590 nm and 529 nm, respectively, using a Cytation^TM^ 5 multimode plate reader. The mitochondrial membrane potential was expressed as the JC-1 aggregate/monomer fluorescence ratio and normalized to the untreated control group [43].

#### 2.3.7. Mitochondrial and Nuclear Fluorescence Staining

To evaluate mitochondrial and nuclear morphology, HGF-1 cells were seeded in 12-well plates at a density of 1 × 10^5^ cells/well and allowed to reach approximately 70% confluence before treatment. The cells were then exposed for 24 h to AgCUR-EtOH NPs and AgCUR-H_2_O NPs at concentrations ranging from 1 to 10 μg/mL. For mitochondrial staining, MitoTracker^TM^ Red CMXRos stock solution (1 mM in DMSO) was diluted in complete culture medium to a final concentration of 300 nM. Cells were incubated with the staining solution for 30 min under standard culture conditions and then washed with fresh medium to remove excess dye. Subsequently, cells were fixed with 4% paraformaldehyde for 10 min at room temperature and washed thoroughly with PBS. Nuclear morphology was assessed by staining with Hoechst 33342, diluted 1:2000 in PBS, for 5–10 min in the dark. The staining solution was then removed, and the cells were washed three times with PBS. Representative fluorescence images were acquired using a Lionheart^TM^ FX automated microscope, and image processing was performed using Gen5^TM^ Microplate Data Collection and Analysis Software. The apoptotic index was estimated from Hoechst-stained images as the percentage of cells showing nuclear condensation and/or fragmentation relative to the total number of analyzed nuclei.

#### 2.3.8. Acridine Orange/Propidium Iodide (AO/PI) Assay

To evaluate apoptotic- and necrotic-like changes in HGF-1 cells, an acridine orange/propidium iodide (AO/PI) dual staining assay was performed. Cells were seeded in 96-well plates at a density of 1 × 10^4^ cells/well and exposed for 24 h to AgCUR-EtOH NPs and AgCUR-H_2_O NPs at concentrations ranging from 1 to 10 μg/mL. Following treatment, 100 μL/well of staining solution prepared in complete culture medium and containing 10 μg/mL AO and 10 μg/mL PI was added to each well. The plates were incubated for 10 min at room temperature in the dark. Representative fluorescence images were then acquired using a Lionheart^TM^ FX automated microscope and analyzed with Gen5^TM^ Microplate Data Collection and Analysis Software. AO/PI-stained cells were qualitatively assessed based on fluorescence pattern and nuclear morphology, with green fluorescence indicating viable or early apoptotic cells and red fluorescence indicating PI-positive cells with compromised membrane integrity. The protocol was carried out in accordance with the method described by Dahma et al. [44].

### 2.4. In Ovo Biocompatibility Screening of CUR-AgNPs

The HET-CAM (Hen’s Egg Test–Chorioallantoic Membrane) assay was used to evaluate the irritant potential and in ovo biocompatibility of AgCUR-H_2_O NPs and AgCUR-EtOH NPs at the level of the chorioallantoic membrane vascular plexus. Fertilized hen eggs (*Gallus gallus domesticus*) were prepared as follows: on the first day of incubation, the eggs were cleaned and placed in an incubator at 37 °C and 60% humidity. On the fourth day of incubation, the eggs were perforated, and approximately 6–7 mL of albumen was removed. On the fifth day, a window was opened in the eggshell to allow visualization of the chorioallantoic membrane; the opening was then covered with adhesive tape, and the eggs were returned to the incubator until the day of the experiment.

The assay was performed on day 10 of incubation. A volume of 600 μL of each sample was applied directly onto the chorioallantoic membrane (CAM). Distilled water was used as the negative control, while 1% sodium dodecyl sulfate (SDS) was used as the positive control. AgCUR-H_2_O NPs and AgCUR-EtOH NPs were tested at a concentration of 10 μg/mL. The experiment was performed in triplicate for each tested sample. The main irritative events monitored were hemorrhage (H), vascular lysis (L), and intravascular coagulation (C). These effects were recorded according to their time of occurrence over a 5 min observation period. Images were acquired before sample application (T_0_) and after 5 min of exposure (T_5_) using a Zeiss Stereo Discovery.V8 stereomicroscope (Zeiss, Göttingen, Germany) equipped with a Zeiss AxioCam 105 color camera.

The irritation score (IS) was calculated according to the following equation:

(3) $$IS=5\times \frac{301-t_{H}}{300}+7\times \frac{301-t_{L}}{300}+9\times \frac{301-t_{C}}{300}$$

where *IS*—represents the irritation score, *t_H_*—the time, in seconds, at which hemorrhage was observed, *t_L_*—the time, in seconds, at which vascular lysis occurred, and *t_C_*—the time, in seconds, at which intravascular coagulation was observed. If no event occurred during the observation period, a value of 300 s was assigned.

According to the commonly used HET-CAM irritation grading system, IS values ranging from 0.0 to 0.9 were classified as non-irritant, values from 1.0 to 4.9 as slight or weak irritant, values from 5.0 to 8.9 as moderately irritant, and values from 9.0 to 21.0 as strongly or severely irritant [26,45].

### 2.5. Statistical Analysis

Quantitative data obtained from the cell-based in vitro assays were expressed as mean values ± standard deviation (SD). For the MTT, NRU, JC-1, and fluorescence-based apoptotic index analyses, experiments were performed in three independent replicates, each carried out in triplicate. Statistical analyses and graphical representations were performed using GraphPad Prism software, version 10.2.3. Statistical analysis was performed using one-way analysis of variance (ANOVA), followed by Dunnett’s multiple comparisons post hoc test to compare each treated group with the untreated control group. Differences were considered statistically significant at *p* < 0.05. The levels of significance were indicated as follows: *p* < 0.05, *p* < 0.01, *p* < 0.001, and *p* < 0.0001. MIC and MBC endpoints were interpreted descriptively, and no inferential statistical comparison was applied to the antibacterial results.

Qualitative microscopy-based evaluations, including bright-field morphology assessment and AO/PI staining, were interpreted descriptively based on representative images. The HET-CAM assay results were expressed as irritation scores (IS) and occurrence times of hemorrhage, vascular lysis, and coagulation, and were interpreted descriptively in comparison with the negative and positive controls.

## 3. Results

### 3.1. Physicochemical Characterization of Green-Synthesized CUR-AgNPs

#### 3.1.1. UV-Visible Spectroscopy

**Figure 1:**
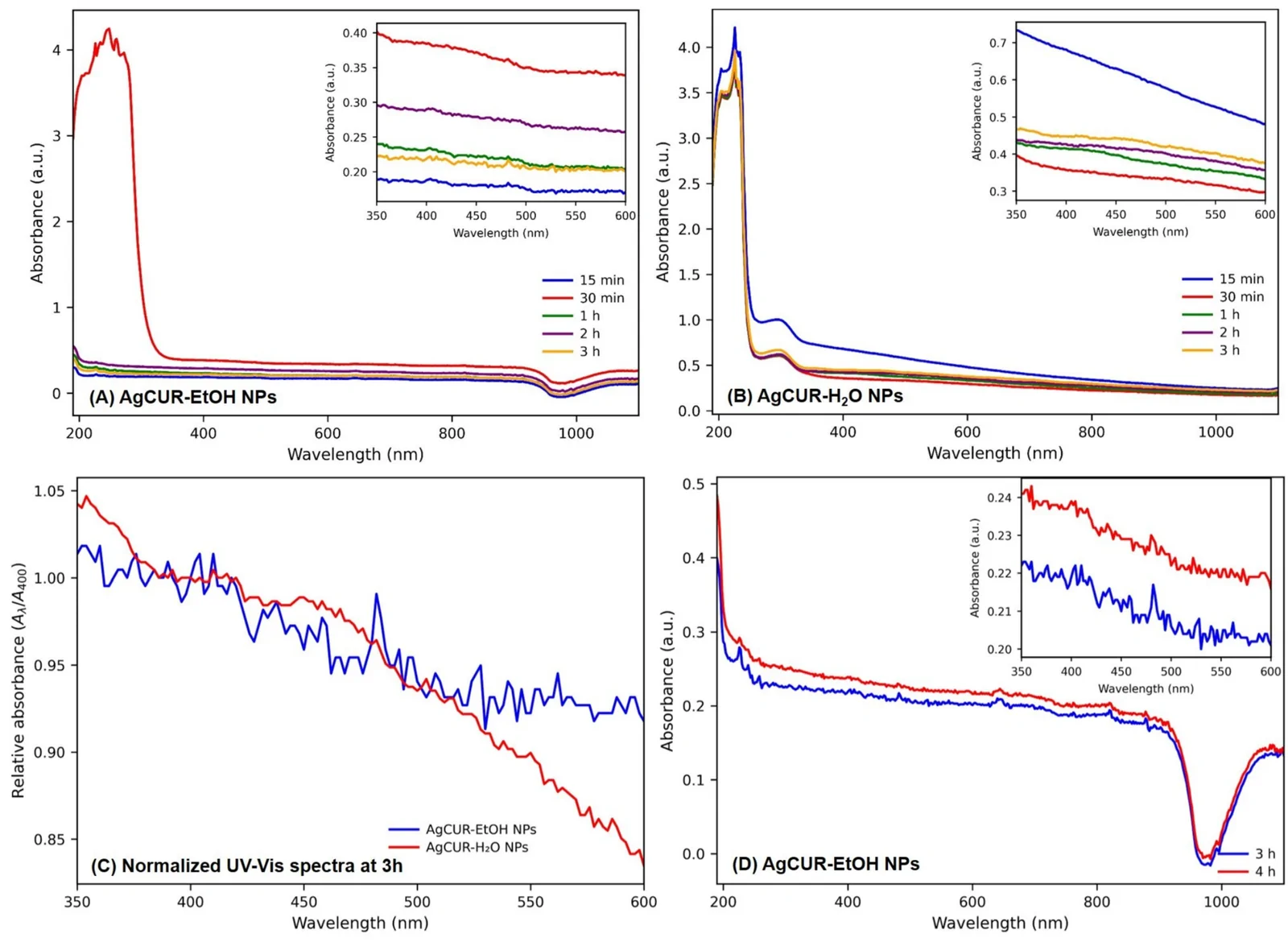
UV–Vis spectral evolution during the green synthesis of CUR-AgNPs. (**A**) Spectra of AgCUR-EtOH NPs recorded after 15 and 30 min and after 1, 2, and 3 h of reaction. Reaction aliquots were diluted 1:1 with ethanol and measured against an ethanol blank. (**B**) Spectra of AgCUR-H_2_O NPs recorded after 15 and 30 min and after 1, 2, and 3 h of reaction. The aliquots collected were diluted 1:20 with distilled water; distilled water was used as the blank. The complete spectra were recorded between 190 and 1100 nm, and the insets highlight the 350–600 nm region. (**C**) Visible-region spectra of AgCUR-EtOH NPs and AgCUR-H_2_O NPs recorded after 3 h and normalized to the corresponding absorbance at 400 nm. Normalization facilitates comparison of relative spectral shape but does not permit quantitative comparison of nanoparticle concentration or synthesis yield. (**D**) UV–Vis spectra of AgCUR-EtOH NPs recorded after 3 and 4 h of reaction before nanoparticle recovery. Reaction aliquots were diluted 1:1 with ethanol and measured against an ethanol blank. Complete spectra are presented over the 190–1100 nm range, while the inset highlights the 350–600 nm visible region. No new absorption band or marked spectral shift was observed during the final hour of reaction.

UV–Vis spectroscopy was used to monitor reaction-associated optical changes during the synthesis of AgCUR-EtOH NPs and AgCUR-H_2_O NPs. The complete spectra recorded between 190 and 1100 nm are presented in Figure 1A,B, while the corresponding insets highlight the 350–600 nm region. Because the two reaction matrices required different diluents and dilution ratios, the spectra were evaluated primarily within each formulation, and raw absorbance intensities were not directly compared between the ethanolic and aqueous systems.

The AgCUR-EtOH NPs spectra showed an intense and complex absorption profile in the ultraviolet region, together with broad, non-resolved absorption extending throughout the visible range (Figure 1A). Within the 350–600 nm interval, absorbance gradually decreased with increasing wavelength, without the appearance of a clearly distinguishable localized surface plasmon resonance maximum. Absolute absorbance values varied non-monotonically between the successive time points, indicating that the optical response was strongly influenced by the ethanolic phytochemical matrix and potentially by light scattering from polydisperse or aggregated structures. Accordingly, these spectra were not interpreted quantitatively in terms of reaction kinetics or nanoparticle concentration.

The AgCUR-H_2_O NPs spectra displayed intense ultraviolet absorption, with a prominent feature at approximately 226 nm, followed by a broad absorption contribution extending into the visible region (Figure 1B). The spectrum recorded after 15 min displayed a markedly higher visible-region absorbance than the subsequent spectra, indicating a non-monotonic early optical response. Because this isolated early signal may reflect transient matrix- and scattering-related effects and/or aliquot heterogeneity, it was not interpreted as evidence of a proportional increase in nanoparticle concentration. From 30 min to 3 h, the absorbance at 400 nm increased progressively from 0.357 to 0.415 after 1 h, 0.427 after 2 h, and 0.448 after 3 h. These changes indicate progressive development of the visible-region optical response within the aqueous reaction system. However, the broad and continuously decreasing profile between 350 and 600 nm did not allow the assignment of a narrow, well-resolved plasmonic maximum.

To compare the relative shapes of the two optical profiles independently of their absolute absorbance scales, the 3 h spectra were normalized to their respective absorbance at 400 nm (Figure 1C). The normalized AgCUR-H_2_O NPs spectrum showed a more pronounced decrease toward longer visible wavelengths, whereas the AgCUR-EtOH NPs spectrum displayed a comparatively flatter profile above approximately 500 nm. These differences support formulation-dependent optical behavior but should not be interpreted as evidence of differences in nanoparticle concentration or synthesis yield.

Because the AgCUR-EtOH synthesis was continued for a total of 4 h before nanoparticle recovery, the spectra recorded after 3 and 4 h were additionally compared in Figure 1D. The two spectra showed closely related overall and visible-region profiles, without the emergence of a new absorption band or a marked spectral shift during the final hour of reaction. A modest increase in the absolute absorbance baseline was observed at 4 h. Overall, the UV–Vis findings indicate reaction-associated optical changes in both systems, particularly within the aqueous formulation.

#### 3.1.2. Hydrodynamic Size Distribution and Zeta Potential

**Figure 2:**
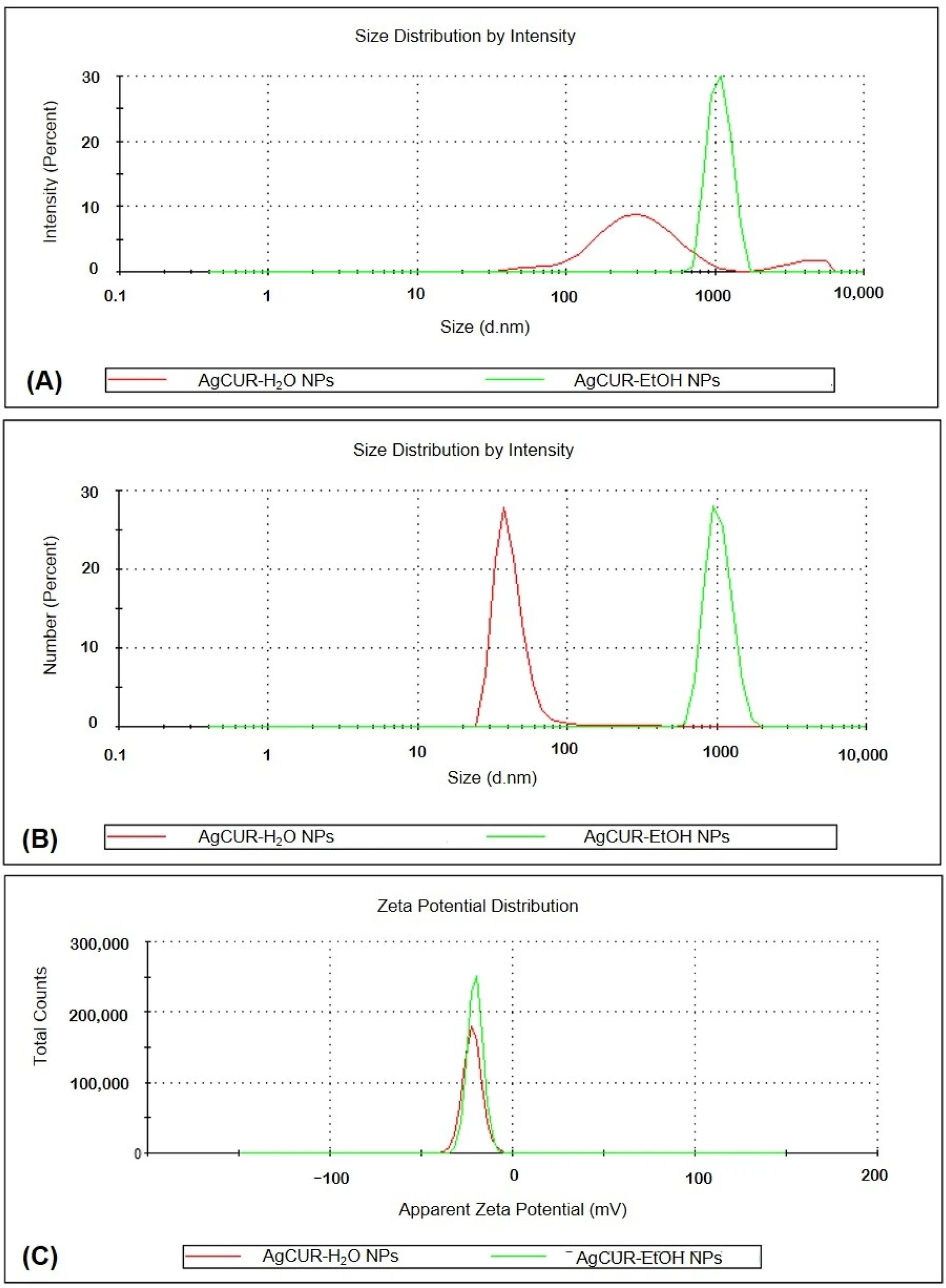
Dynamic light scattering and zeta-potential characterization of the redispersed CUR-AgNP formulations. (**A**) Intensity-weighted hydrodynamic size distributions; (**B**) number-weighted hydrodynamic size distributions; and (**C**) apparent zeta-potential distributions of AgCUR-H_2_O NPs redispersed in distilled water and AgCUR-EtOH NPs redispersed in ethanol. Measurements were performed at 30 °C. The results describe the dispersion behavior of the final dried powders after redispersion in their respective solvent systems.

Dynamic light scattering analysis showed broad hydrodynamic size distributions for both redispersed CUR-AgNP formulations under their respective solvent-specific measurement conditions. AgCUR-H_2_O NPs, redispersed in distilled water, displayed a Z-average hydrodynamic diameter of 264 nm and a PDI of 0.416. AgCUR-EtOH NPs, redispersed in ethanol, exhibited a markedly larger Z-average diameter of 1742 nm and a PDI of 0.500 (Table 1 and Figure 2). The PDI values indicate heterogeneous and polydisperse dispersions in both cases. The high Z-average value obtained for AgCUR-EtOH NPs indicates a pronounced contribution from large scattering entities, consistent with the presence of agglomerated particles and/or phytochemical matrix-associated structures in the ethanolic redispersion. By comparison, the AgCUR-H_2_O NPs dispersion showed a smaller hydrodynamic size under the tested aqueous conditions, although its PDI also confirmed a broad particle population.

The intensity- and number-weighted hydrodynamic size distributions are presented in Figure 2A,B, whereas the apparent zeta-potential distributions are shown in Figure 2C. Both formulations exhibited negative apparent zeta-potential values in their respective dispersants. AgCUR-H_2_O NPs showed an apparent zeta potential of −21.8 ± 0.8 mV in distilled water, whereas AgCUR-EtOH NPs showed a value of −20.4 ± 0.6 mV in ethanol. These findings indicate a negative electrokinetic potential for both redispersed formulations under the tested conditions. Nevertheless, because the measurements were performed in different dispersing media, the absolute hydrodynamic diameters and zeta-potential magnitudes should not be interpreted as directly equivalent indicators of their intrinsic colloidal stability.

#### 3.1.3. X-Ray Diffraction Analysis

**Figure 3:**
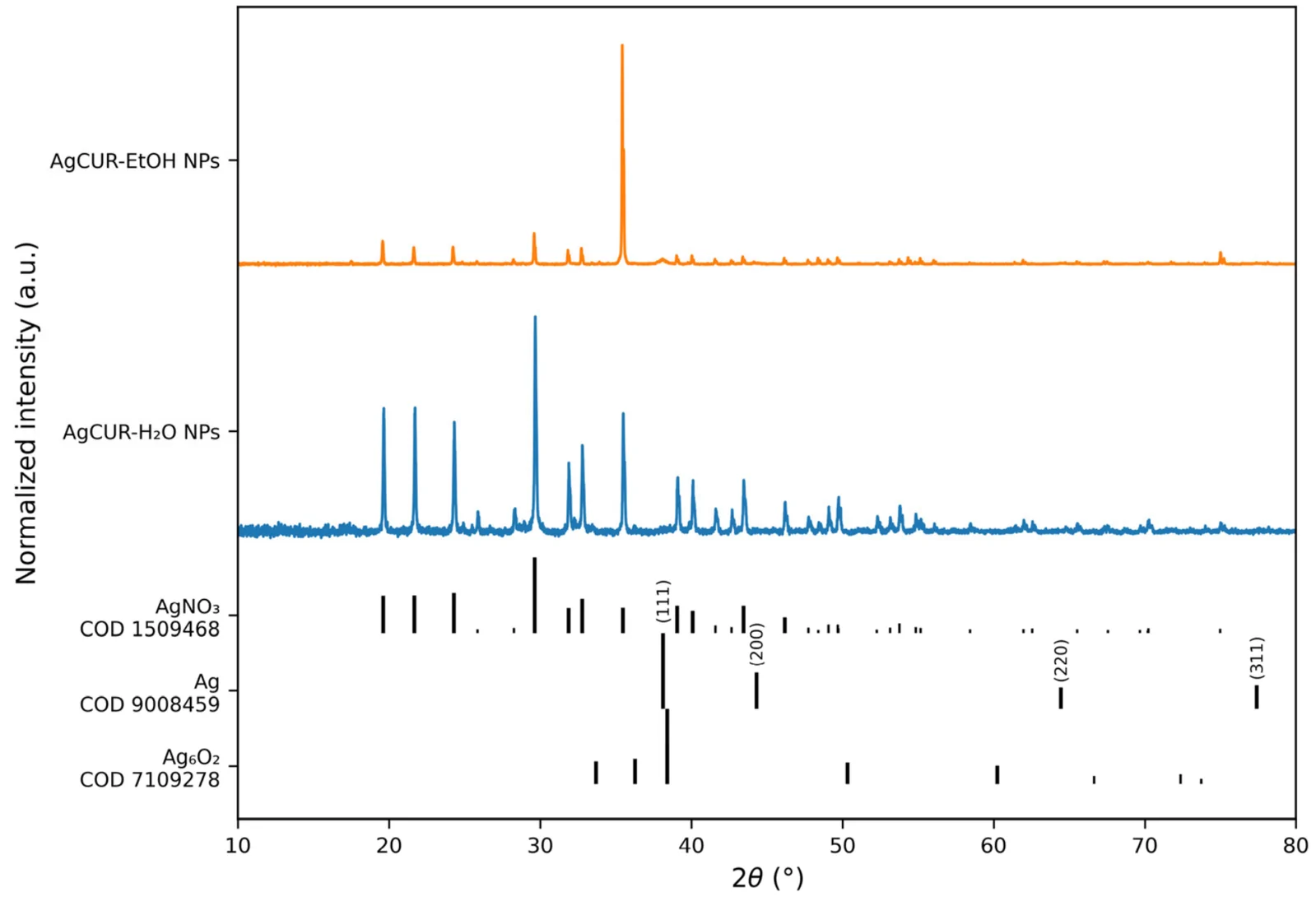
Powder X-ray diffraction profiles of AgCUR-EtOH NPs and AgCUR-H_2_O NPs, together with reference stick patterns for crystalline AgNO_3_ (COD 1509468), face-centered cubic metallic Ag (COD 9008459), and Ag_6_O_2_ (COD 7109278). The principal crystallographic planes of metallic Ag are indicated. Each experimental profile was baseline-shifted, normalized independently to its maximum intensity, and vertically offset for visual clarity. Reference reflections are presented as black vertical lines. No smoothing or interpolation was applied to the experimental data. The profiles were dominated by reflections consistent with crystalline AgNO_3_, while weaker reflections were compatible with metallic Ag and a possible minor oxidized silver phase. Normalized diffraction intensities should not be interpreted as quantitative phase fractions.

The powder X-ray diffraction profiles of AgCUR-EtOH NPs and AgCUR-H_2_O NPs are presented in Figure 3 together with the corresponding reference stick patterns. Both formulations displayed multiple narrow and well-defined diffraction reflections, demonstrating the presence of crystalline constituents in the dried samples. Most of the intense experimental reflections showed close correspondence with the reference pattern of crystalline AgNO_3_ (COD 1509468). Representative peaks were detected at approximately 2θ = 19.6°, 21.7°, 24.3°, 29.6°, 31.9°, 32.8°, and 35.5° in both formulations. Additional reflections occurring in the approximately 39–44° region also corresponded to reflections of the AgNO_3_ reference phase. These findings indicate that residual crystalline silver nitrate was retained in both dried CUR-AgNP formulations.

Differences were observed in the relative intensities of several reflections. In particular, AgCUR-EtOH NPs showed a highly intense and narrow peak at approximately 2θ = 35.4°, which was also detected in the AgCUR-H_2_O NPs profile but at a lower relative intensity. Because the two experimental patterns were normalized independently and diffraction intensity may be influenced by crystallite orientation and sample preparation, the relative intensity difference cannot be interpreted as a direct quantitative measure of the amount of AgNO_3_ present in each formulation.

Weaker features were additionally observed close to 2θ = 38.1°, 44.3°, 64.4°, and 77.4°. These positions are compatible with the (111), (200), (220), and (311) planes, respectively, of face-centered cubic metallic Ag (COD 9008459). The comparatively low intensity and partial overlap of these reflections with those of the other crystalline phases prevented unequivocal quantitative evaluation; nevertheless, their presence supports the formation of a crystalline metallic Ag contribution in both formulations.

Additional low-intensity reflections showed partial correspondence with the Ag_6_O_2_ reference pattern (COD 7109278), particularly in the regions around 2θ = 33–36°, 60°, 66–67°, and 72°. However, because these reflections were weak and overlapped with peaks assigned to AgNO_3_ and metallic Ag, the presence of a minor oxidized silver phase should be considered tentative.

Scherrer analysis of four comparatively intense and resolved AgNO_3_ reflections yielded apparent mean coherent crystallite sizes of 82.0 ± 3.1 nm for AgCUR-EtOH NPs and 68.2 ± 5.6 nm for AgCUR-H_2_O NPs, expressed as mean ± standard deviation across the four selected reflections. These values refer exclusively to the residual crystalline AgNO_3_ phase and were not corrected for instrumental broadening. Crystallite-size estimation was not performed for metallic Ag because its comparatively weak reflections partially overlapped with those of AgNO_3_ and Ag_6_O_2_.

Overall, the XRD profiles demonstrated that both CUR-AgNP formulations contained multiple silver-associated crystalline phases. The patterns were dominated by reflections consistent with residual crystalline AgNO_3_, whereas weaker features supported the presence of face-centered cubic metallic Ag and suggested a possible minor oxidized silver contribution. Quantitative phase fractions were not calculated because of extensive peak overlap, potential preferred orientation, and the absence of full-pattern structural refinement.

#### 3.1.4. Fourier-Transform Infrared Spectroscopy

**Figure 4:**
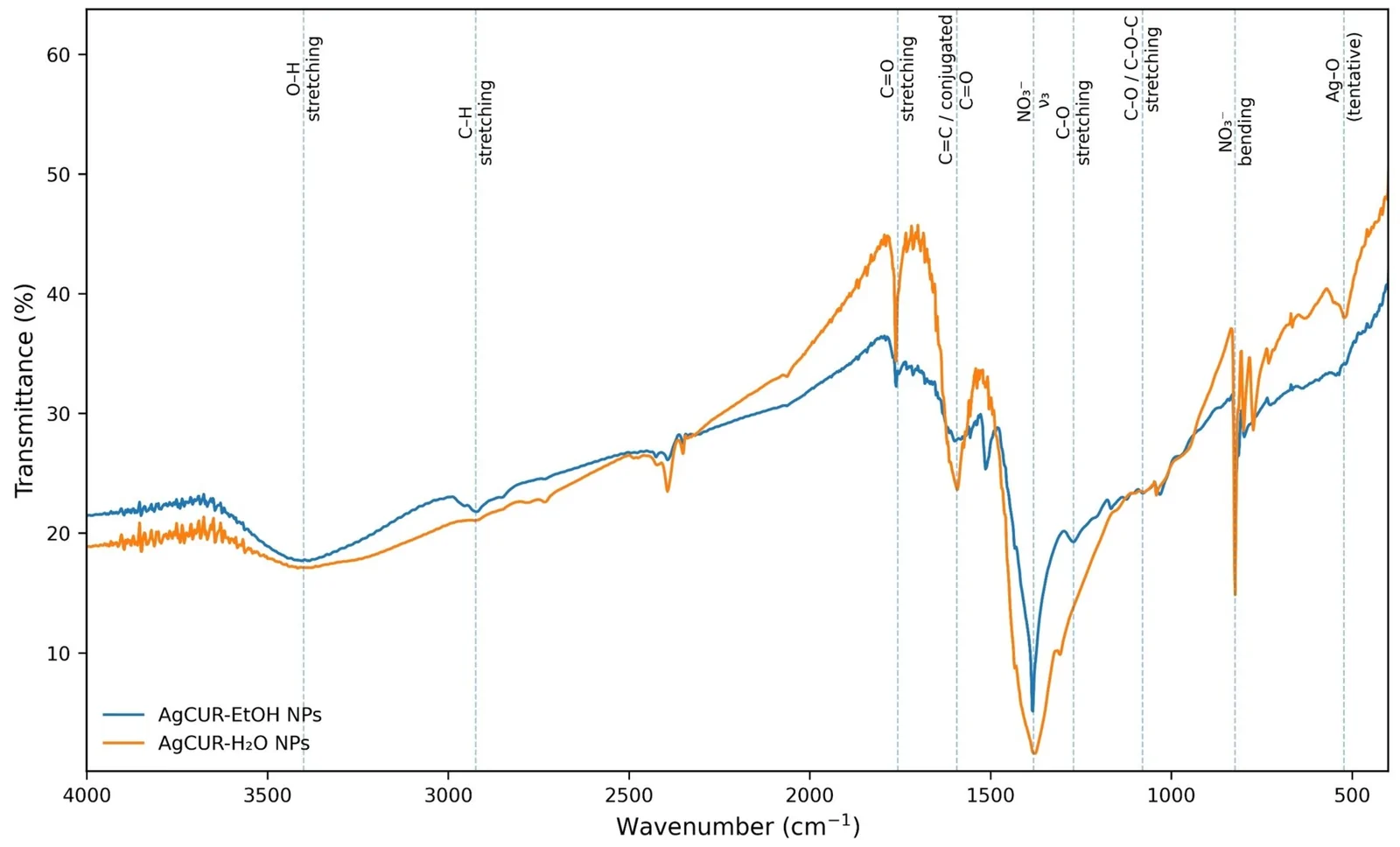
Fourier-transform infrared spectra of the dried AgCUR-EtOH NPs and AgCUR-H_2_O NPs formulations recorded in transmittance mode over the 4000–400 cm^−1^ range using KBr pellets. The principal tentative vibrational assignments are indicated. Both formulations exhibited extract-derived organic functional-group contributions together with prominent nitrate-associated bands at approximately 1380 and 824 cm^−1^. The weak feature near 523 cm^−1^ in AgCUR-H_2_O NPs was tentatively assigned to an Ag–O-related vibration. The spectra are presented as recorded, without normalization or smoothing.

The FTIR spectra of the dried AgCUR-EtOH NPs and AgCUR-H_2_O NPs formulations are presented in Figure 4, while the principal absorption bands and their tentative vibrational assignments are summarized in Table 2. Both formulations exhibited complex spectral profiles containing contributions attributable to extract-derived organic constituents and nitrate-containing inorganic species.

The AgCUR-EtOH NPs spectrum displayed a broad absorption band centered at approximately 3400 cm^−1^, assigned to O–H stretching vibrations of hydrogen-bonded hydroxyl groups and adsorbed moisture. A distinct band was observed at approximately 2924 cm^−1^ and was attributed to aliphatic C–H stretching. Additional features were detected at approximately 1755–1761, 1591, and 1510 cm^−1^. The band near 1591 cm^−1^ was assigned to aromatic C=C stretching and/or conjugated carbonyl vibrations, whereas the feature near 1510 cm^−1^ was consistent with an aromatic skeletal vibration. The approximately 1760 cm^−1^ signal may contain contributions from carbonyl-containing extract constituents and/or nitrate-related combination bands and was therefore not assigned exclusively to a single functional group.

In the fingerprint region of AgCUR-EtOH NPs, bands were identified at approximately 1271, 1165, and 1032 cm^−1^. These features are compatible with phenolic or enolic C–O and C–O–C stretching vibrations associated with curcuminoids, polyphenols, carbohydrates, and other oxygen-containing extract constituents. However, nitrate-related vibrational contributions may also occur within this region, particularly near approximately 1030–1040 cm^−1^. The AgCUR-H_2_O NPs spectrum also showed a broad O–H stretching contribution centered around 3400 cm^−1^. Absorption features were observed at approximately 1760 and 1593 cm^−1^, whereas the bands around 1510 and 1270 cm^−1^ were less clearly resolved than in AgCUR-EtOH NPs. A weak feature was detected around 1038 cm^−1^. Overall, the organic fingerprint of AgCUR-H_2_O NPs appeared less differentiated than that of the ethanolic-extract-mediated formulation. Both formulations exhibited a particularly intense absorption band at approximately 1380 cm^−1^, with minima near 1383 cm^−1^ for AgCUR-EtOH NPs and approximately 1377–1379 cm^−1^ for AgCUR-H_2_O NPs. A second intense band was observed at approximately 824 cm^−1^ in both spectra. These features were assigned predominantly to nitrate vibrational modes and indicate the presence of residual nitrate-containing species in the dried formulations. The attribution is consistent with the residual crystalline AgNO_3_ phase identified by XRD.

A weak low-wavenumber band was additionally detected at approximately 523 cm^−1^ in AgCUR-H_2_O NPs. This feature may be compatible with an Ag–O lattice vibration; however, because low-wavenumber signals may contain overlapping inorganic and organic contributions, this assignment was considered tentative.

Overall, the spectra demonstrate that extract-derived organic functional groups remained associated with both dried CUR-AgNP formulations. AgCUR-EtOH NPs exhibited a more clearly resolved organic fingerprint, particularly through the bands assigned to aliphatic C–H, aromatic C=C, aromatic skeletal, and phenolic/enolic C–O vibrations. In contrast, the AgCUR-H_2_O NPs spectrum contained fewer clearly differentiated organic bands. Nevertheless, because the original CUR-EtOH and CUR-H_2_O extracts were not analyzed by FTIR under identical experimental conditions, specific spectral shifts and definitive solvent-dependent coordination mechanisms could not be established.

#### 3.1.5. Electron Microscopy Analysis

**Figure 5:**
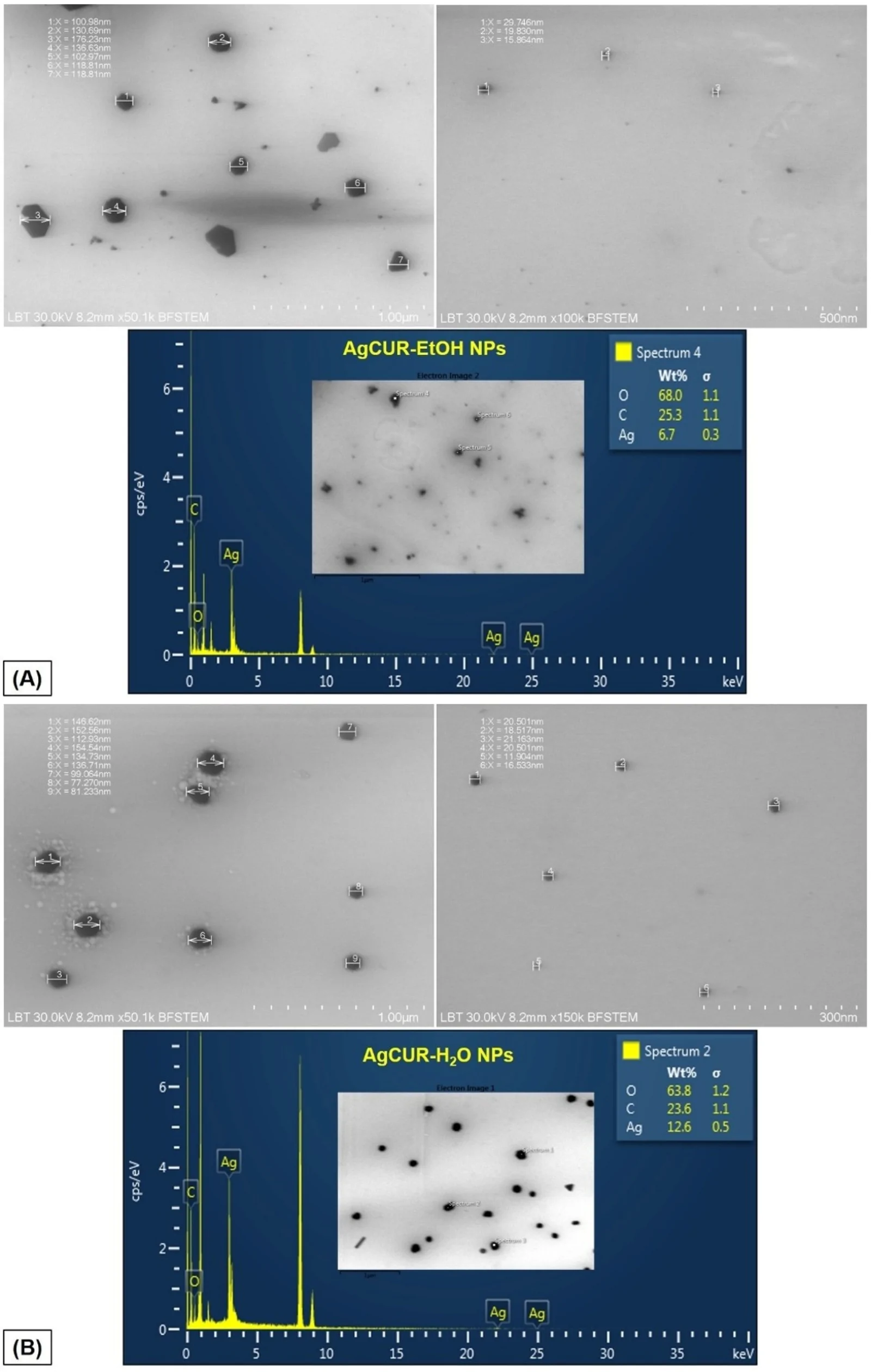
Representative TEM micrographs and EDX spectra of green-synthesized CUR-AgNPs: (**A**) AgCUR-EtOH NPs and (**B**) AgCUR-H_2_O NPs. TEM images show polydisperse nanoparticles and larger irregular structures distributed on the grid surface. EDX spectra confirmed the presence of silver in both samples, together with C and O signals attributed to the organic matrix and/or carbon-coated support grid.

TEM analysis was further performed to assess the morphology and size range of the green-synthesized AgCUR-EtOH NPs and AgCUR-H_2_O NPs. As shown in Figure 5A, AgCUR-EtOH NPs displayed a heterogeneous morphology, with nanoscale particles and larger irregular structures distributed on the grid surface. Based on the representative micrographs, the particle size ranged approximately from 15 to 175 nm, indicating a polydisperse nanoparticle population. The presence of smaller particles was also observed at higher magnification, although partial aggregation and overlapping organic matrix residues were evident in some regions.

In the case of AgCUR-H_2_O NPs, TEM images revealed a similarly polydisperse morphology, with particles ranging approximately from 15 to 150 nm (Figure 5B). Compared with the ethanolic extract-mediated sample, the aqueous extract-mediated nanoparticles showed a more clearly distinguishable population of small particles in the lower nanometer range, together with larger particles or aggregates distributed across the grid. These observations suggest that both extract types were able to mediate the formation of nanoscale silver-containing particles, although with different degrees of dispersion and morphological heterogeneity.

EDX analysis confirmed the presence of elemental silver in both samples. Representative local EDX spectra showed Ag signals corresponding to 6.7 wt% in the analyzed AgCUR-EtOH NPs region and 12.6 wt% in the AgCUR-H_2_O NPs region. In addition to Ag, intense C and O signals were detected, which may be attributed to the organic phytochemical matrix associated with the biosynthesized nanoparticles, as well as to contributions from the carbon-coated support grid and the additional carbon coating used during sample preparation. Overall, the TEM and EDX results support the successful formation of Ag-containing nanoparticles using both ethanolic and aqueous *Curcuma longa* extracts.

### 3.2. Antibacterial Activity of CUR-AgNPs

The antibacterial activity of AgCUR-EtOH NPs and AgCUR-H_2_O NPs was evaluated against *S. mutans*, *S. aureus*, and *S. oralis* by determining their MIC and MBC values. The results, expressed as µg/mL of total dried CUR-AgNP formulation, are presented in Table 3. Both formulations exhibited strain-dependent antibacterial activity. *S. mutans* was the most susceptible microorganism, with MIC values of 9 µg/mL for AgCUR-EtOH NPs and 7 µg/mL for AgCUR-H_2_O NPs. The corresponding MBC values were 88 and 62 µg/mL, respectively. Thus, both formulations inhibited *S. mutans* growth at concentrations below 10 µg/mL, although substantially higher concentrations were required to achieve a bactericidal effect.

Intermediate susceptibility was observed for *S. aureus*. AgCUR-EtOH NPs exhibited MIC and MBC values of 168 and 266 µg/mL, respectively, whereas AgCUR-H_2_O NPs showed lower corresponding values of 138 and 192 µg/mL.

*S. oralis* was the least susceptible of the three tested bacterial strains. MIC values of 235 and 157 µg/mL were obtained for AgCUR-EtOH NPs and AgCUR-H_2_O NPs, respectively, while the corresponding MBC values were 462 and 385 µg/mL.

Overall, AgCUR-H_2_O NPs consistently exhibited lower MIC and MBC values than AgCUR-EtOH NPs against all three bacterial strains, indicating a stronger antibacterial effect under the applied experimental conditions. The difference between the two formulations was particularly evident against *S. oralis*, for which the aqueous-extract-mediated formulation showed a considerably lower MIC.

### 3.3. In Vitro Cytocompatibility Profile of CUR-AgNPs

#### 3.3.1. Dose-Dependent Effects of AgCUR-NPs on HGF-1 Cell Viability

**Figure 6:**
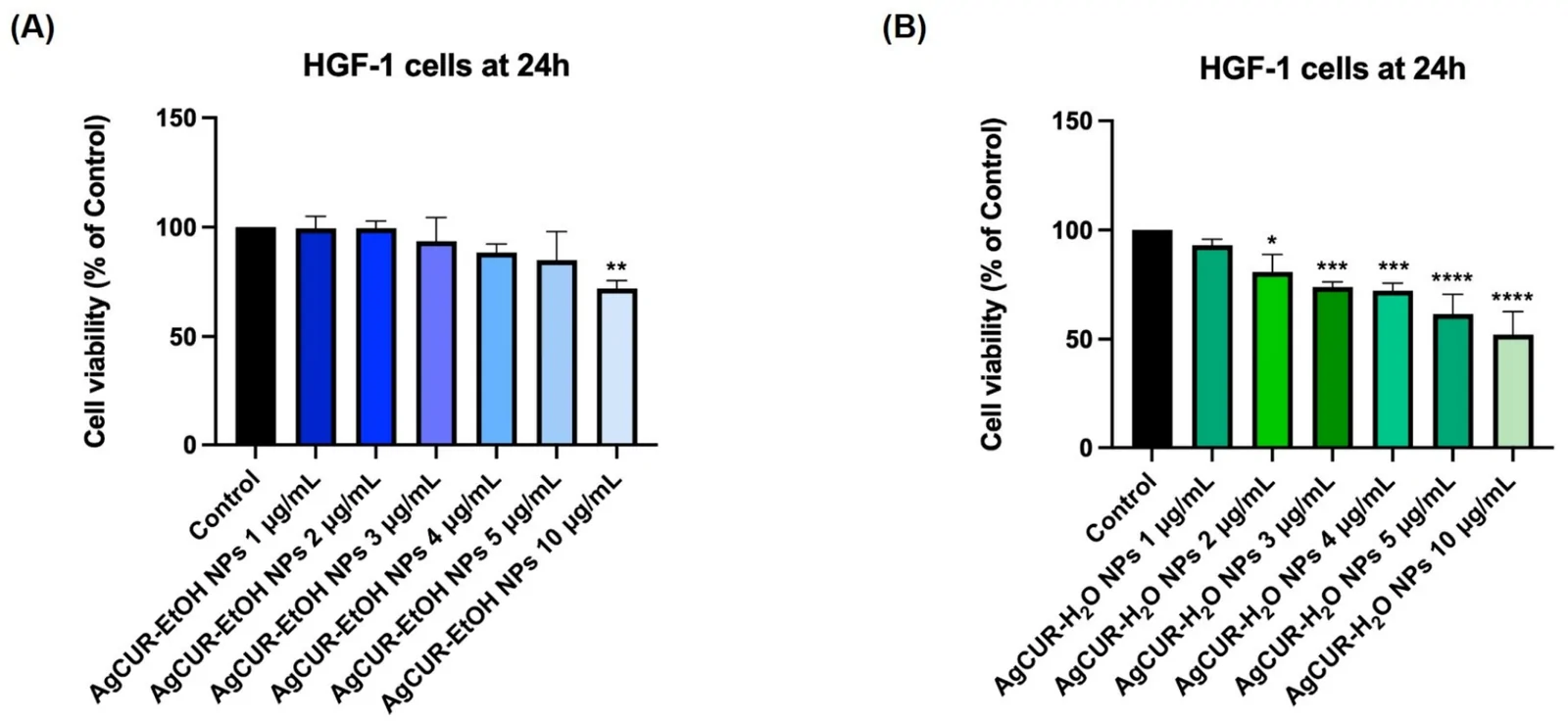
The viability of HGF-1 cells after 24 h of exposure to: (**A**) AgCUR-EtOH NPs, (**B**) AgCUR-H_2_O NPs at concentrations of 1–10 µg/mL. Results are expressed as percentages (%) normalized to the untreated control group. Data are presented as mean values ± standard deviation (SD) from three independent experiments, each performed in triplicate. Statistical differences between treated groups and the control were assessed using one-way ANOVA followed by Dunnett’s multiple comparisons post hoc test (* *p* < 0.05; ** *p* < 0.01; *** *p* < 0.001; **** *p* < 0.0001).

The effect of AgCUR-EtOH NPs and AgCUR-H_2_O NPs on HGF-1 cell viability was evaluated after 24 h of exposure to concentrations ranging from 1 to 10 μg/mL (Figure 6). Overall, both nanoparticle formulations induced a concentration-dependent reduction in cell viability, although the response differed between the two samples. AgCUR-EtOH NPs preserved cell viability close to that of the untreated control at the lower tested concentrations, with cell viability values of 99.46%, 99.62%, and 93.57% at 1, 2, and 3 μg/mL, respectively. At higher concentrations, viability decreased to 88.38% at 4 μg/mL, 84.98% at 5 μg/mL, and 71.88% at 10 μg/mL. In contrast, AgCUR-H_2_O NPs produced a more pronounced decrease in HGF-1 cell viability across the tested concentration range. Cell viability decreased from 93.03% at 1 μg/mL to 80.96%, 73.99%, 72.36%, 61.56%, and 52.14% at 2, 3, 4, 5, and 10 μg/mL, respectively. These data indicate that AgCUR-H_2_O NPs exerted a stronger concentration-dependent reduction in HGF-1 cell viability compared with AgCUR-EtOH NPs, while AgCUR-EtOH NPs maintained higher cytocompatibility, particularly at concentrations up to 5 μg/mL.

#### 3.3.2. Morphological Response of HGF-1 Cells to CUR-AgNPs

**Figure 7:**
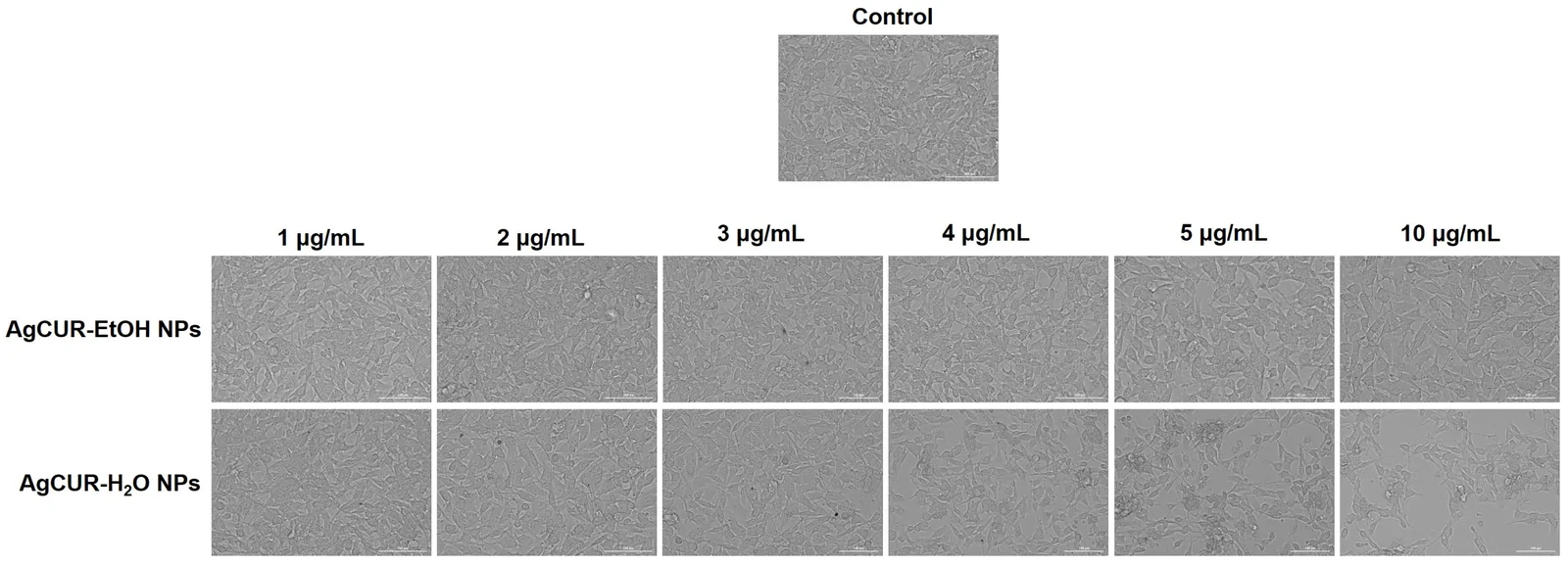
Representative bright-field images illustrating morphological changes in HGF-1 cells after 24 h of exposure to AgCUR-EtOH NPs and AgCUR-H_2_O NPs at concentrations of 1–10 µg/mL. The scale bar indicates 100 µm.

Bright-field microscopy was used to further evaluate the cytocompatibility profile of AgCUR-EtOH NPs and AgCUR-H_2_O NPs by assessing HGF-1 cell morphology after 24 h of treatment (Figure 7). Cells were exposed to increasing concentrations of each nanoparticle formulation, ranging from 1 to 10 μg/mL. In the case of AgCUR-EtOH NPs, only a slight reduction in cell confluence was observed, without major morphological alterations across the tested concentration range. The cells largely maintained their typical fibroblast-like morphology, particularly at low and intermediate concentrations. In contrast, AgCUR-H_2_O NPs induced a more pronounced reduction in cell confluence, accompanied by visible morphological alterations, including cell rounding and shrinkage, especially at higher concentrations of 5 and 10 μg/mL. These observations are consistent with the MTT results and further support the stronger concentration-dependent impact of AgCUR-H_2_O NPs on HGF-1 cell viability.

#### 3.3.3. Lysosomal Neutral Red Uptake Response of HGF-1 Cells to CUR-AgNPs

**Figure 8:**
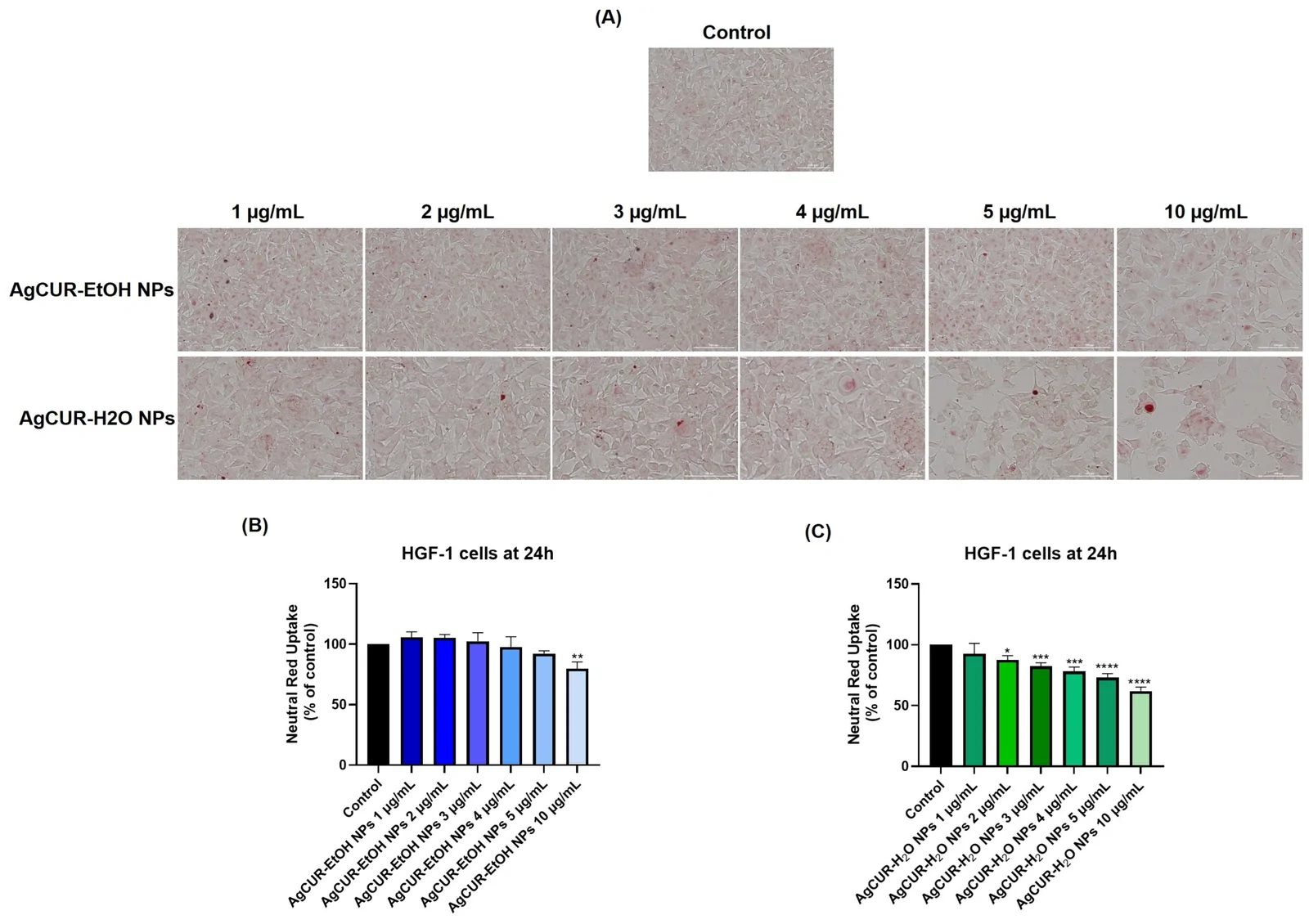
Effect of AgCUR-EtOH NPs and AgCUR-H_2_O NPs on lysosomal neutral red uptake in HGF-1 cells after 24 h of exposure. (**A**) Representative bright-field images showing intracellular neutral red uptake. (**B**) NRU percentages following treatment with AgCUR-EtOH NPs. (**C**) NRU percentages following treatment with AgCUR-H_2_O NPs. Results are expressed as percentages (%) normalized to the untreated control group. Data are presented as mean values ± standard deviation (SD) from three independent experiments, each performed in triplicate. Statistical differences between treated groups and the control were evaluated using one-way ANOVA followed by Dunnett’s multiple comparisons post hoc test (* *p* < 0.05; ** *p* < 0.01; *** *p* < 0.001; **** *p* < 0.0001). The scale bar indicates 100 µm.

The NRU assay was performed to further evaluate the impact of AgCUR-EtOH NPs and AgCUR-H_2_O NPs on lysosomal integrity in HGF-1 cells after 24 h of exposure (Figure 8). Microscopic evaluation of neutral red staining generally indicated preserved lysosomal dye uptake in cells treated with AgCUR-EtOH NPs, with only a slight reduction observed at the highest tested concentration of 10 μg/mL. In contrast, AgCUR-H_2_O NPs induced a more visible reduction in neutral red uptake, particularly from 5 μg/mL onward.

Quantitative analysis confirmed these observations. For AgCUR-EtOH NPs, NRU values remained close to or slightly above the control level at low concentrations, with values of 105.71%, 105.15%, and 102.43% at 1, 2, and 3 μg/mL, respectively. At 4 μg/mL, NRU was 97.52%, followed by a decrease to 92.16% at 5 μg/mL and 79.87% at 10 μg/mL (Figure 8B). In the case of AgCUR-H_2_O NPs, a concentration-dependent decrease in NRU was observed across the tested range, with values of 92.57%, 87.46%, 82.33%, 78.01%, 72.98%, and 61.99% at 1, 2, 3, 4, 5, and 10 μg/mL, respectively (Figure 8C). Overall, these results indicate that AgCUR-EtOH NPs better preserved lysosomal neutral red uptake, whereas AgCUR-H_2_O NPs exerted a stronger concentration-dependent impact on lysosomal function.

#### 3.3.4. Mitochondrial Membrane Potential Response of HGF-1 Cells to CUR-AgNPs

**Figure 9:**
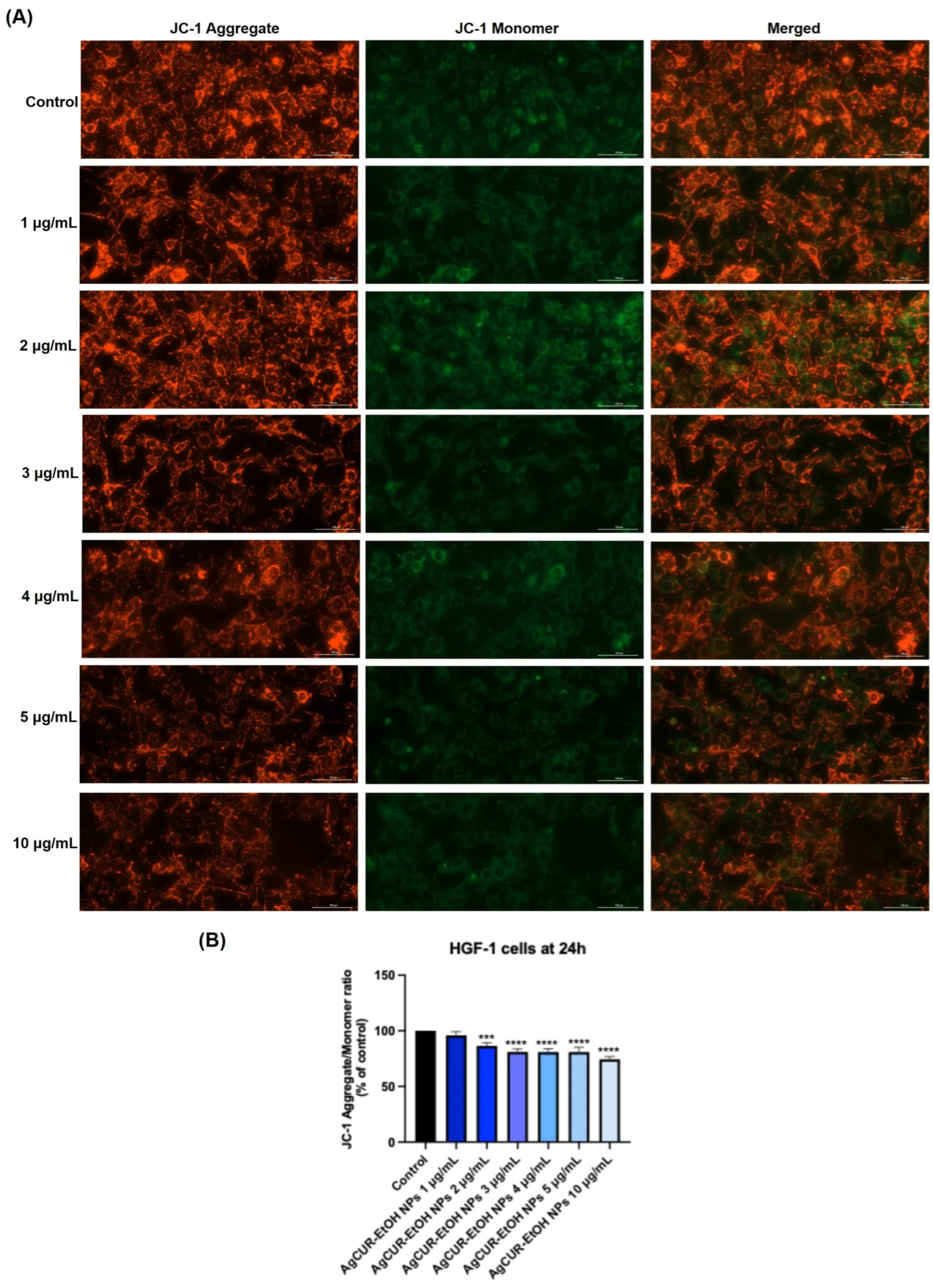
Effect of AgCUR-EtOH NPs on mitochondrial membrane potential (ΔΨm) in HGF-1 cells after 24 h of exposure. (**A**) Representative JC-1 staining images illustrating mitochondrial status; red fluorescence corresponds to JC-1 aggregates in polarized mitochondria, while green fluorescence corresponds to JC-1 monomers associated with mitochondrial depolarization. Images were captured at 20× magnification; the scale bar indicates 100 µm. (**B**) Quantitative analysis of the JC-1 aggregate/monomer fluorescence ratio, expressed as a percentage relative to the untreated control group. Data are presented as mean values ± standard deviation (SD). Statistical differences between treated groups and the control were evaluated using one-way ANOVA followed by Dunnett’s multiple comparisons post hoc test (*** *p* < 0.001; **** *p* < 0.0001).

JC-1 staining was used to further evaluate the impact of AgCUR-EtOH NPs and AgCUR-H_2_O NPs on mitochondrial membrane potential in HGF-1 cells after 24 h of exposure. In the case of AgCUR-EtOH NPs, fluorescence microscopy showed a predominance of red fluorescence across the tested concentration range, indicating that mitochondrial polarization was largely preserved (Figure 9A). Quantitative analysis of the JC-1 aggregate/monomer ratio showed a moderate concentration-associated decrease, with values of 95.89%, 86.46%, 81.07%, 80.88%, 80.88%, and 74.41% at 1, 2, 3, 4, 5, and 10 μg/mL, respectively (Figure 9B).

**Figure 10:**
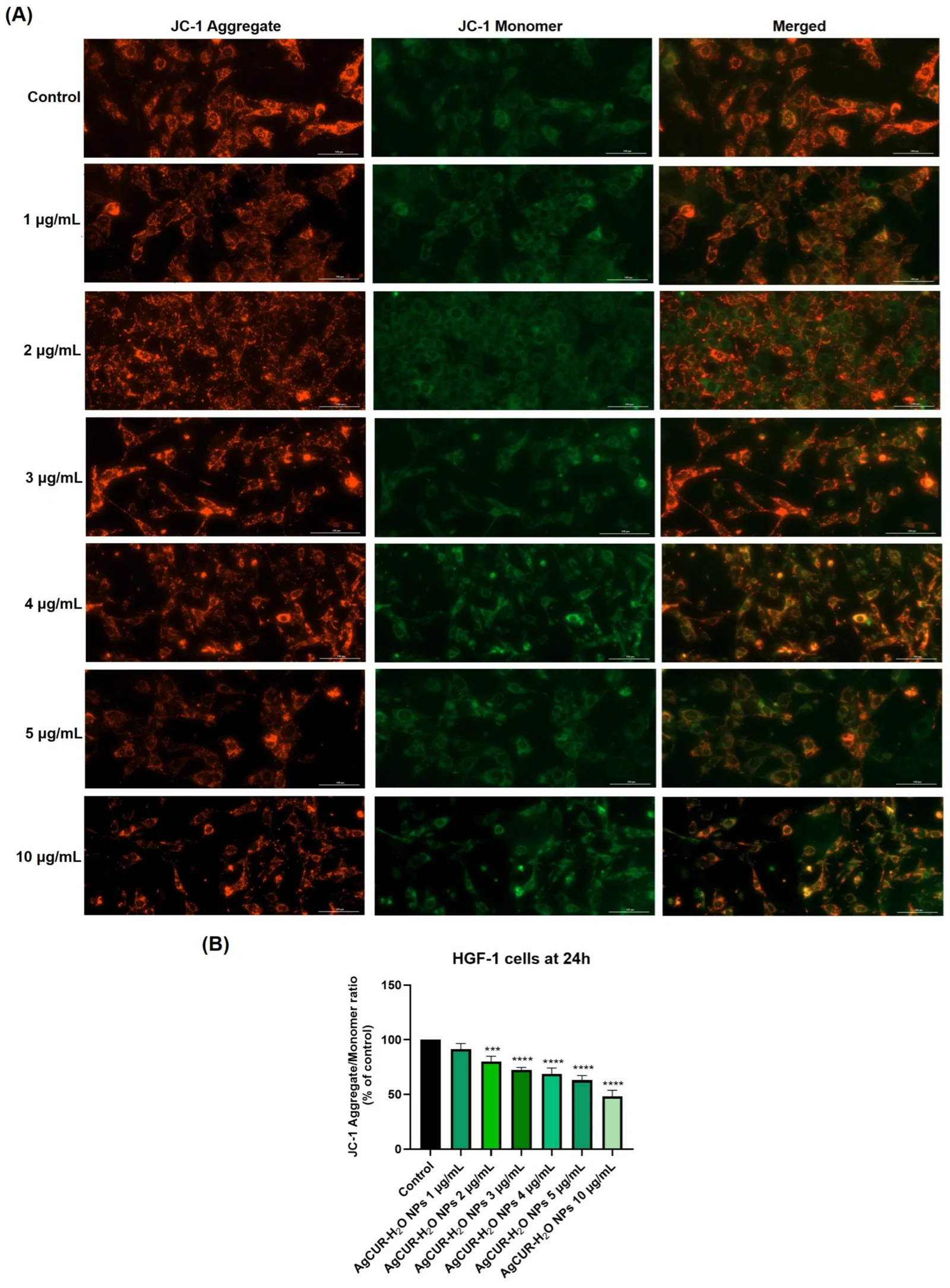
Effect of AgCUR-H_2_O NPs on mitochondrial membrane potential (ΔΨm) in HGF-1 cells after 24 h of exposure. (**A**) Representative JC-1 staining images illustrating mitochondrial status; red fluorescence corresponds to JC-1 aggregates in polarized mitochondria, while green fluorescence corresponds to JC-1 monomers associated with mitochondrial depolarization. Images were captured at 20× magnification; the scale bar indicates 100 µm. (**B**) Quantitative analysis of the JC-1 aggregate/monomer fluorescence ratio, expressed as a percentage relative to the untreated control group. Data are presented as mean values ± standard deviation (SD). Statistical differences between treated groups and the control were evaluated using one-way ANOVA followed by Dunnett’s multiple comparisons post hoc test (*** *p* < 0.001; **** *p* < 0.0001).

For AgCUR-H_2_O NPs, microscopic evaluation revealed a gradual reduction in red fluorescence and a concomitant increase in green fluorescence, particularly from 4 μg/mL onward, suggesting a more pronounced mitochondrial depolarization (Figure 10A). Quantitative analysis confirmed a concentration-dependent decrease in the JC-1 aggregate/monomer ratio, with values of 91.28%, 80.22%, 72.28%, 68.82%, 63.11%, and 48.33% at 1, 2, 3, 4, 5, and 10 μg/mL, respectively (Figure 10B). Overall, AgCUR-EtOH NPs exerted a milder impact on mitochondrial membrane potential, whereas AgCUR-H_2_O NPs induced a stronger concentration-dependent mitochondrial response, consistent with the MTT and NRU results.

#### 3.3.5. Mitochondrial and Nuclear Alterations in HGF-1 Cells After CUR-AgNPs Exposure

**Figure 11:**
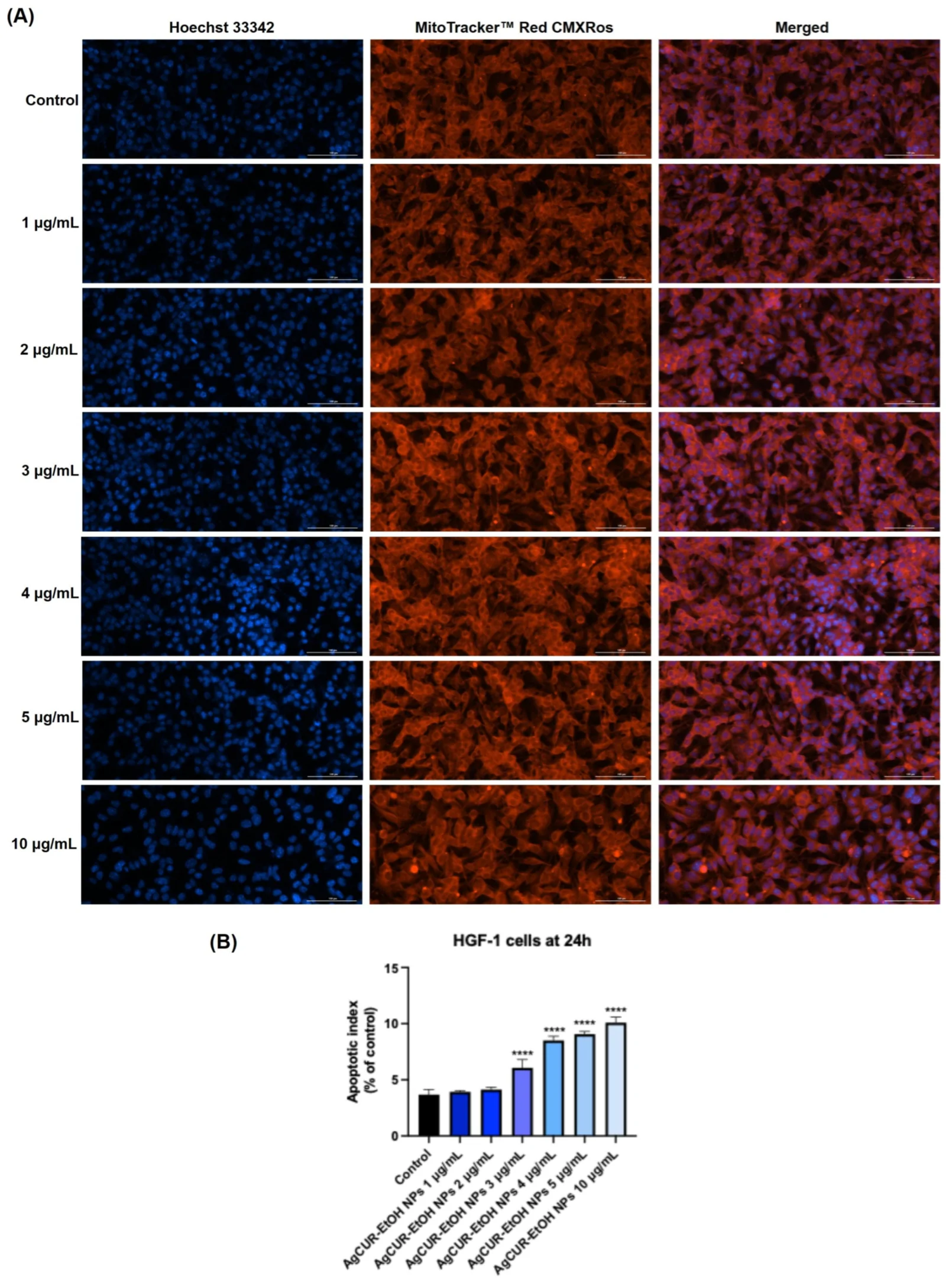
Effect of AgCUR-EtOH NPs on mitochondrial and nuclear morphology in HGF-1 cells after 24 h of exposure. (**A**) Representative fluorescence images obtained after MitoTracker^TM^ Red CMXRos and Hoechst 33342 staining, highlighting mitochondrial organization and nuclear morphology. Images were captured at 20× magnification; the scale bar indicates 100 µm. (**B**) Apoptotic index (%), calculated based on Hoechst-stained nuclei and expressed as mean values ± standard deviation (SD). Statistical differences between treated groups and the control were evaluated using one-way ANOVA followed by Dunnett’s multiple comparisons post hoc test (**** *p* < 0.0001).

**Figure 12:**
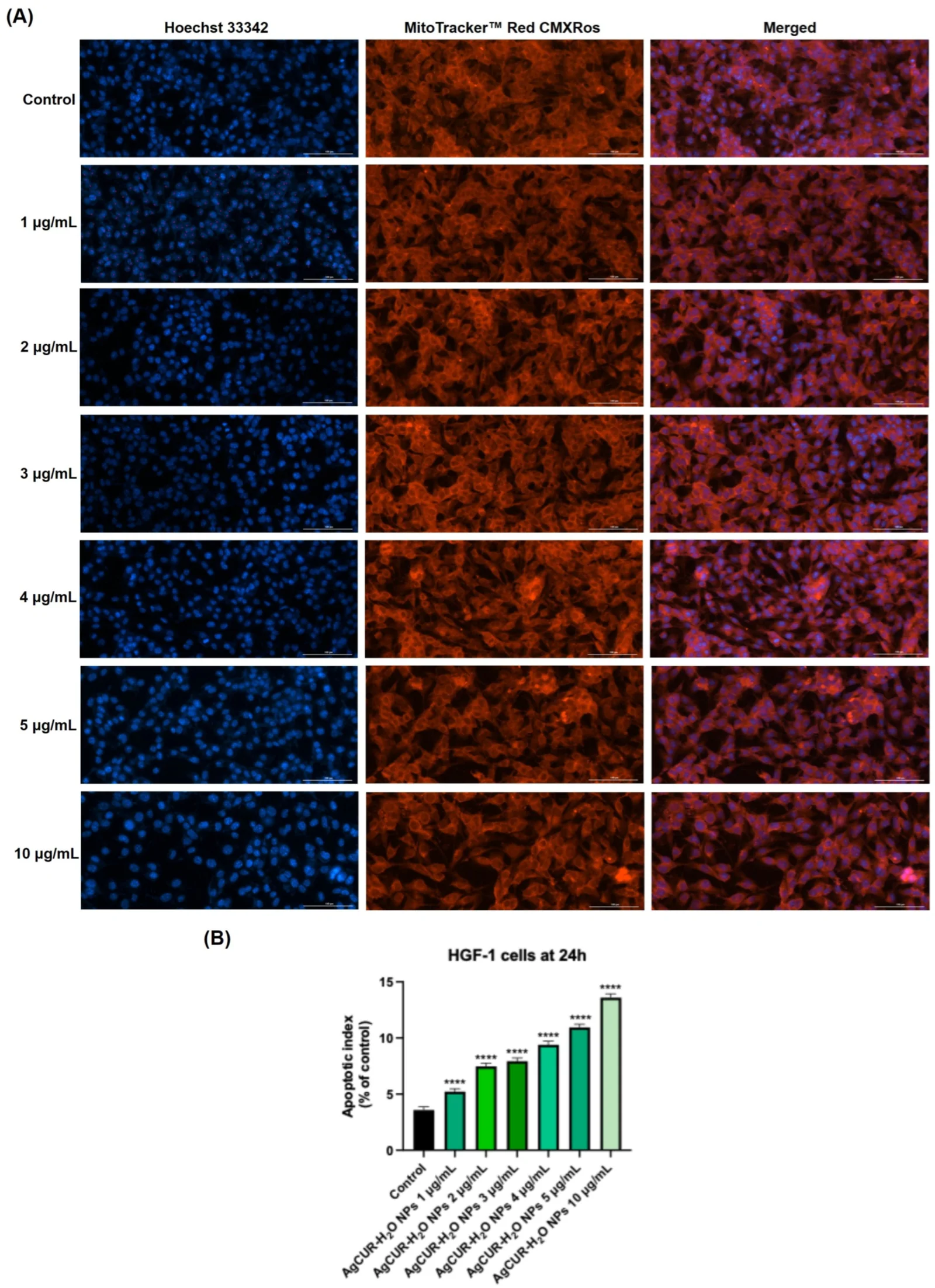
Effect of AgCUR-H_2_O NPs on mitochondrial and nuclear morphology in HGF-1 cells after 24 h of exposure. (**A**) Representative fluorescence images obtained after MitoTracker^TM^ Red CMXRos and Hoechst 33342 staining, highlighting mitochondrial organization and nuclear morphology. Images were captured at 20× magnification; the scale bar indicates 100 µm. (**B**) Apoptotic index (%), calculated based on Hoechst-stained nuclei and expressed as mean values ± standard deviation (SD). Statistical differences between treated groups and the control were evaluated using one-way ANOVA followed by Dunnett’s multiple comparisons post hoc test (**** *p* < 0.0001).

Fluorescence staining with MitoTracker^TM^ Red CMXRos and Hoechst 33342 was used to further evaluate mitochondrial organization and nuclear morphology in HGF-1 cells after 24 h of treatment with AgCUR-EtOH NPs and AgCUR-H_2_O NPs (Figure 11 and Figure 12). In the case of AgCUR-EtOH NPs, the nuclei remained mostly uniform and regular, comparable to those observed in untreated control cells, while the mitochondrial network appeared largely preserved across the tested concentration range (Figure 11A). At the highest concentration tested, 10 μg/mL, slight chromatin condensation and minor changes in fluorescence intensity were observed. Quantitative analysis showed a gradual increase in the apoptotic index, from approximately 3.93% at 1 μg/mL to 10.10% at 10 μg/mL (Figure 11B).

In contrast, cells treated with AgCUR-H_2_O NPs showed more evident mitochondrial and nuclear alterations, particularly from 4 μg/mL onward (Figure 12A). These changes included increased nuclear condensation and altered mitochondrial fluorescence patterns, becoming more pronounced at higher concentrations. Consistently, the apoptotic index increased from approximately 5.22% at 1 μg/mL to 13.60% at 10 μg/mL (Figure 12B). Overall, these findings indicate that AgCUR-EtOH NPs better preserved mitochondrial and nuclear morphology, whereas AgCUR-H_2_O NPs induced a stronger concentration-associated cellular stress response, in agreement with the MTT, NRU, and JC-1 results.

#### 3.3.6. AO/PI-Based Evaluation of Apoptotic- and Necrotic-like Changes in HGF-1 Cells

**Figure 13:**
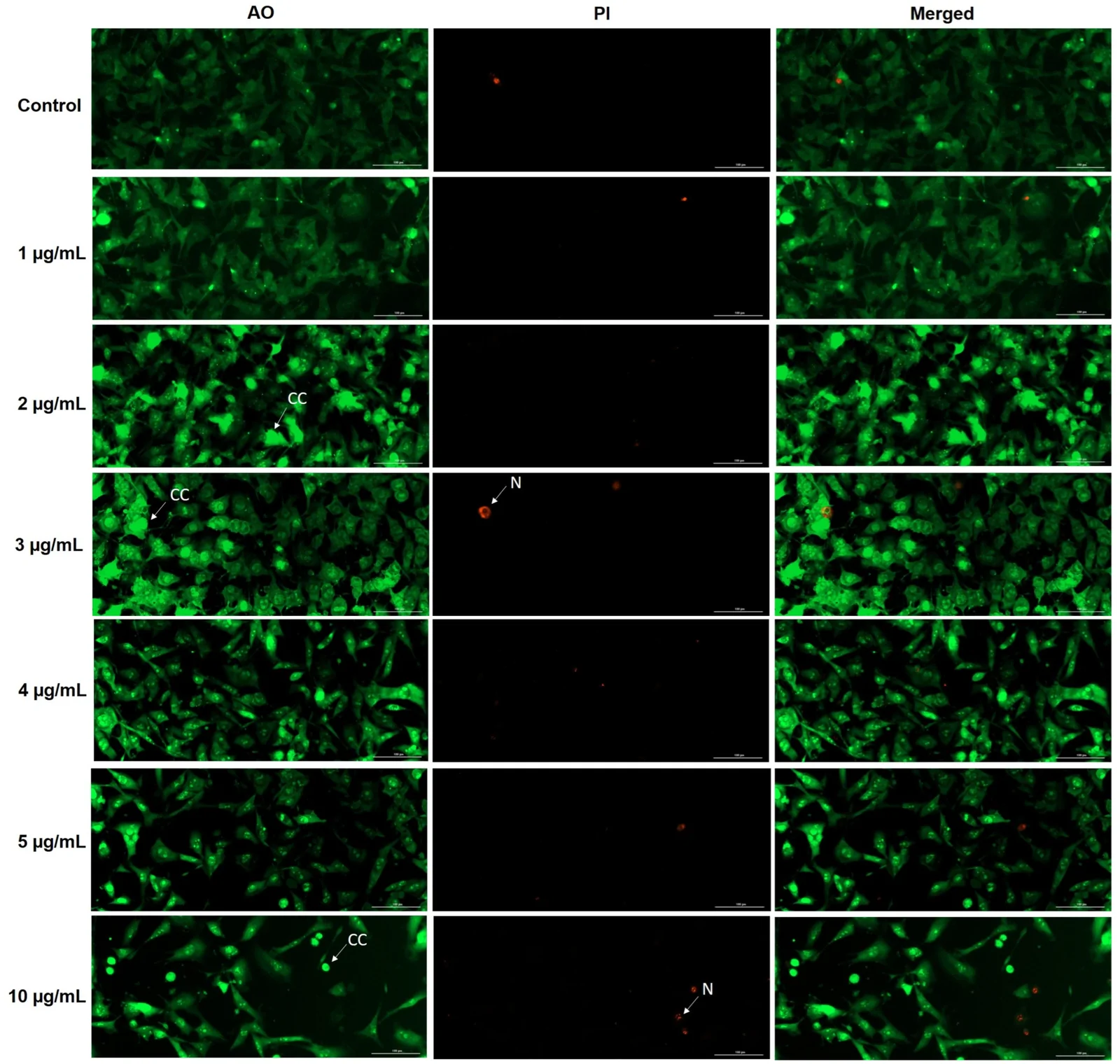
Representative fluorescence images of HGF-1 cells stained with acridine orange/propidium iodide (AO/PI) after 24 h of exposure to AgCUR-EtOH NPs at concentrations of 1–10 µg/mL. Images were acquired at 20× magnification; the scale bar indicates 100 µm. CC: chromatin condensation; N: PI-positive cells with compromised membrane integrity.

AO/PI dual staining was used to further assess apoptotic- and necrotic-like changes in HGF-1 cells after 24 h of exposure to AgCUR-EtOH NPs and AgCUR-H_2_O NPs. Following treatment with AgCUR-EtOH NPs, fluorescence microscopy showed a predominance of green-stained cells across the tested concentration range, indicating that most cells maintained membrane integrity (Figure 13). Mild apoptotic-like nuclear changes, mainly represented by chromatin condensation, were observed starting from 2 μg/mL and became more evident at the highest tested concentration of 10 μg/mL. Sporadic PI-positive cells, visible as red fluorescence, were also detected; however, their presence remained comparable to that observed in the untreated control.

**Figure 14:**
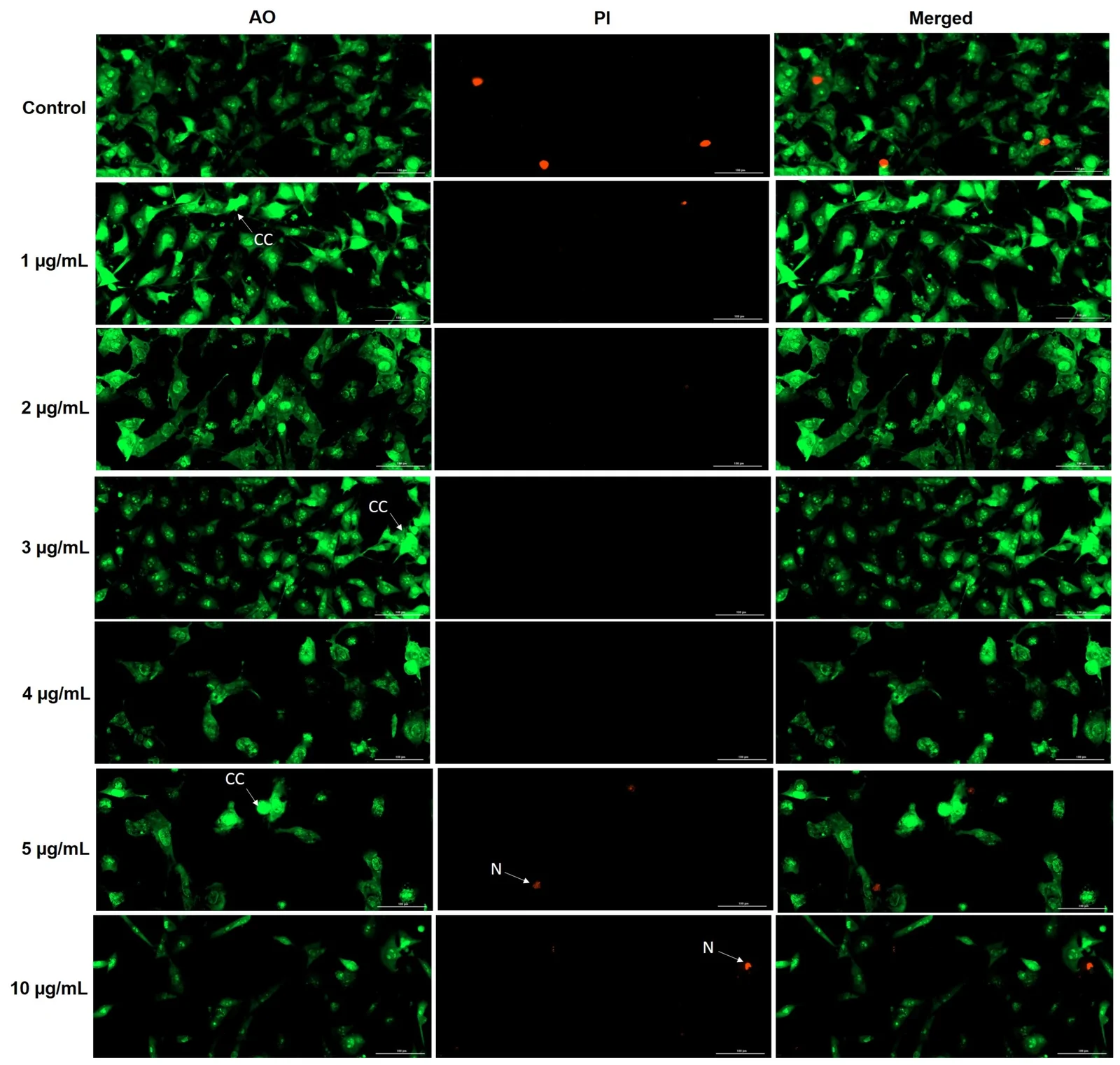
Representative fluorescence images of HGF-1 cells stained with acridine orange/propidium iodide (AO/PI) after 24 h of exposure to AgCUR-H_2_O NPs at concentrations of 1–10 µg/mL. Images were acquired at 20× magnification; the scale bar indicates 100 µm. CC: chromatin condensation; N: PI-positive cells with compromised membrane integrity.

In the case of AgCUR-H_2_O NPs, a predominance of green fluorescence was also observed, suggesting the persistence of a viable cell population after 24 h of exposure (Figure 14). Nevertheless, mild apoptotic-like features were visible from the lowest tested concentration, together with a gradual reduction in cell confluence across the concentration range. PI-positive cells were observed mainly at 5 and 10 μg/mL, but their frequency remained limited and comparable to the control. Overall, AO/PI staining supported the cytocompatibility profile observed in the previous assays, indicating that AgCUR-EtOH NPs induced only mild nuclear alterations, whereas AgCUR-H_2_O NPs produced more evident concentration-associated apoptotic-like changes without a marked increase in necrotic cell features.

### 3.4. In Ovo Biocompatibility Profile of CUR-AgNPs

**Figure 15:**
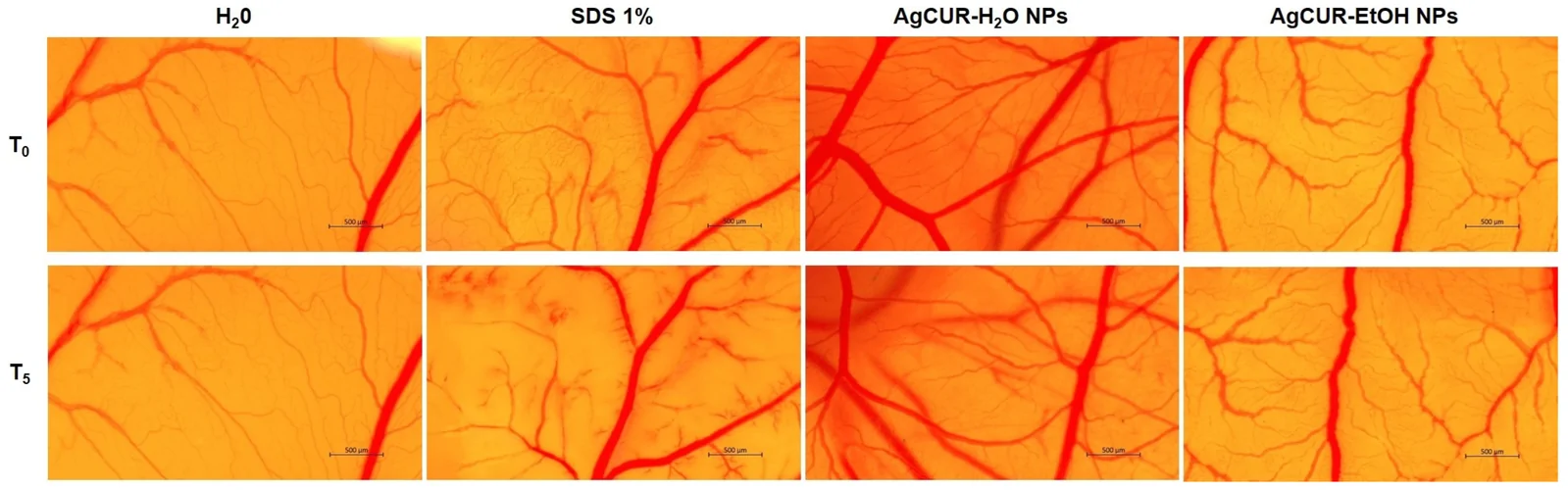
HET-CAM evaluation of the irritant potential of AgCUR-H_2_O NPs and AgCUR-EtOH NPs. Representative stereomicroscopic images of chorioallantoic membranes treated with distilled water as a negative control, 1% SDS as a positive control, and the tested CUR-AgNP formulations. Images were acquired before sample application (T_0_) and after 5 min of exposure (T_5_).

The in ovo biocompatibility of AgCUR-H_2_O NPs and AgCUR-EtOH NPs was evaluated using the HET-CAM assay, with distilled water as the negative control and 1% SDS as the positive control. As shown in Figure 15, application of the tested CUR-AgNPs onto the chorioallantoic membrane did not induce major vascular alterations during the 5 min observation period. The vascular architecture remained largely preserved, without evident hemorrhage, vascular lysis, or extensive coagulation. A slight tendency toward intravascular coagulation was observed for AgCUR-EtOH NPs, but this effect appeared limited and did not resemble the marked irritative response induced by the positive control.

In contrast, the 1% SDS solution produced severe vascular alterations, including hemorrhage, vascular lysis, and coagulation, confirming the responsiveness of the HET-CAM model. Quantitative analysis further supported the low irritant potential of the tested nanoparticles. The irritation scores were 0.07 for distilled water, 19.73 for 1% SDS, 0.69 for AgCUR-H_2_O NPs, and 1.06 for AgCUR-EtOH NPs (Table 4). According to the standard HET-CAM irritation grading criteria, AgCUR-H_2_O NPs, with an IS value of 0.69, were classified as non-irritant, whereas AgCUR-EtOH NPs, with an IS value of 1.06, were classified as a slight or weak irritant. The AgCUR-EtOH NPs score was situated at the lower boundary of the slight-irritation category and remained markedly closer to the negative-control value of 0.07 than to the positive-control value of 19.73. Thus, AgCUR-EtOH NPs produced only a very low acute vascular irritation response under the tested conditions.

## 4. Discussion

Nanotechnology represents an advanced strategy for improving the delivery of therapeutic agents to target tissues through the use of drug-loaded nanoparticles. Due to their small size and high surface-to-volume ratio, nanoparticles increase treatment efficacy by improving biodistribution, stability, and tissue compatibility, while reducing adverse effects and drug resistance [46]. Silver nanoparticles (AgNPs) have attracted considerable interest for biomedical and dental applications due to their antimicrobial properties, high surface reactivity, and potential incorporation into wound dressings, restorative materials, prosthetic devices, endodontic materials, implant coatings, and other antimicrobial biomaterials. However, their biological performance is strongly influenced by synthesis route, particle size, surface chemistry, stabilizing matrix, and dose; therefore, cytocompatibility and irritation screening remain essential before considering further biomedical use [1,47,48].

In the present study, turmeric powder-derived *Curcuma longa* ethanolic and aqueous extracts were used as reducing and stabilizing matrices for AgNP biosynthesis. The interpretation of these systems should consider the solvent-dependent differences previously reported for the corresponding extracts. In a recent study from our group, CUR-EtOH showed a higher extraction yield, antioxidant potential, total phenolic content, and a richer targeted polyphenolic profile than CUR-H_2_O, particularly in terms of p-coumaric and ferulic acid derivatives [20]. These differences indicate that the two extracts represent distinct phytochemical matrices, which may influence Ag^+^ reduction, nanoparticle stabilization, optical behavior, and subsequent biological response.

UV–Vis spectroscopy was used as a preliminary tool to monitor reaction progression and AgNP formation, and the resulting spectral profiles highlighted the matrix-dependent character of the optical response. A narrow and clearly resolved plasmonic maximum was not observed for either formulation, particularly for AgCUR-EtOH NPs, whose spectral profile was strongly influenced by the broad absorption background of the ethanolic phytochemical matrix. AgCUR-EtOH NPs displayed intense absorption in the UV region and a strong contribution around 420–424 nm, overlapping with the spectral region generally associated with AgNP surface plasmon resonance. Consequently, after AgNO_3_ addition, the AgCUR-EtOH NPs spectra showed a broad UV–visible profile rather than a clearly resolved plasmonic band. This does not exclude nanoparticle formation, but indicates that, in this system, UV–Vis spectroscopy should be interpreted mainly as supportive evidence of reaction progression. The stronger optical background of the ethanolic system may be related to its previously reported phenolic-rich composition [20].

In the aqueous system, AgCUR-H_2_O NPs showed a broad visible-region shoulder between approximately 380 and 500 nm, with a gradual increase in absorbance around 400 nm during the 3 h reaction period. This profile is consistent with the expected surface plasmon resonance region of plant-mediated AgNPs, commonly reported around 400–450 nm, depending on particle size, shape, dispersion state, and stabilizing matrix. Comparable UV–Vis features have been reported for *Curcuma longa*-related AgNP systems, including the absorption peak around 415 nm described by Shameli et al., the 420–425 nm range reported by Singh et al., and the 350–430 nm region observed by Venkatadri et al. [15,49,50]. In plant-mediated nanoparticle systems, overlapping absorption from extract-derived chromophores, particle polydispersity, aggregation, and light scattering may broaden or partially obscure the localized surface plasmon resonance response. The more progressive increase in visible-region absorbance observed for AgCUR-H_2_O NPs between 30 min and 3 h suggests a more readily detectable evolution of the aqueous reaction system. Nevertheless, because different matrix-specific measurement conditions were required, the normalized comparison was restricted to relative spectral shape and cannot establish differences in silver conversion efficiency, nanoparticle concentration, or synthesis yield. Recent papers of plant-extract-mediated AgNP synthesis also emphasize that phytochemical composition influences nanoparticle formation, size distribution, morphology, and stabilization efficiency [14]. Therefore, the UV–Vis interpretation was kept qualitative, particularly because different dilution ratios were required to obtain readable spectra.

DLS and zeta-potential analyses complemented the optical characterization by providing information on the hydrodynamic behavior and apparent electrokinetic properties of the dried CUR-AgNP powders after redispersion. AgCUR-H_2_O NPs redispersed in distilled water showed a Z-average diameter of 264 nm and a PDI of 0.416, whereas AgCUR-EtOH NPs redispersed in ethanol exhibited a substantially larger Z-average value of 1742 nm and a PDI of 0.500. The resulting hydrodynamic diameter may include the silver-containing core, the extract-derived surface layer, the associated solvent shell, and any particle–particle or particle–matrix aggregates. Moreover, the intensity-weighted DLS signal is highly sensitive to larger scattering entities, which can disproportionately increase the Z-average value even when smaller particles remain present in the dispersion [51,52,53].

Accordingly, the Z-average value of 1742 nm obtained for AgCUR-EtOH NPs should not be interpreted as the size of the individual silver nanoparticles. Rather, it reflects the presence of micron-scale agglomerates and/or phytochemical matrix-associated clusters under the tested ethanolic redispersion conditions. This interpretation is consistent with the broad optical profile of the ethanolic system and with the larger irregular structures and partial aggregation observed in the TEM micrographs. However, the absolute hydrodynamic sizes of the two formulations must be compared cautiously because the powders were redispersed in different media. Distilled water and ethanol differ in viscosity, dielectric properties, refractive index, solvation capacity, and their interactions with extract-derived surface molecules. These solvent-dependent factors can affect particle wetting, corona conformation, aggregation, diffusion, and the calculated hydrodynamic diameter. Therefore, the lower Z-average observed for AgCUR-H_2_O NPs cannot be attributed exclusively to an intrinsically superior colloidal stability of the aqueous-extract-mediated nanoparticles.

Both formulations exhibited negative apparent zeta-potential values under their corresponding measurement conditions: −21.8 ± 0.8 mV for AgCUR-H_2_O NPs in distilled water and −20.4 ± 0.6 mV for AgCUR-EtOH NPs in ethanol. The negative electrokinetic potential may reflect the presence of ionizable or adsorbed oxygen-containing phytochemical groups associated with the particle surface. Nevertheless, the numerical similarity of the two values should not be interpreted as evidence of equivalent surface charge density or colloidal stability. Zeta potential is dependent not only on the particle surface but also on dispersant viscosity, dielectric constant, conductivity, ionic environment, and the structure of the electrical double layer. Direct cross-solvent comparison of the absolute values is therefore inherently limited [52,54,55,56]. Medium-dependent changes in apparent surface charge and aggregation behavior have also been demonstrated for AgNPs under different solvent, pH, ionic-strength, and electrolyte conditions [57,58]. The negative zeta-potential values may provide a degree of electrostatic repulsion under the tested conditions, but the DLS profiles indicate that this contribution was insufficient to prevent the formation of broad particle populations and large associated structures, particularly in the ethanolic redispersion. In plant-mediated nanoparticle systems, stabilization may involve a combination of electrostatic effects, steric interactions, extract-derived organic coatings, hydrogen bonding, and phytochemical bridging.

The post-synthesis processing procedure should also be considered. DLS and zeta-potential analyses were performed after centrifugation, drying, manual grinding, and redispersion of the nanoparticle powders. Consequently, the measurements characterize the behavior of the final redispersed formulations rather than the original colloidal state present during synthesis. Drying may promote irreversible particle–particle or particle–matrix associations that cannot be completely reversed upon redispersion.

XRD analysis provided complementary structural information regarding the crystalline composition of the two CUR-AgNP formulations. Weak reflections compatible with the (111), (200), (220), and (311) planes of face-centered cubic Ag supported the formation of a crystalline metallic silver fraction during the extract-mediated reduction process. This observation complements the UV–Vis findings and the TEM/EDX confirmation of silver-containing nanoscale structures. Nevertheless, the diffraction profiles were dominated by reflections assigned to crystalline AgNO_3_. The presence of this precursor-related crystalline phase indicates that Ag^+^ reduction was incomplete and/or that a fraction of unreacted AgNO_3_ remained associated with the formulations after synthesis. In the present protocol, the samples were recovered by centrifugation and subsequently dried, without an additional washing step. Consequently, retained silver nitrate may have remained entrapped within the extract-derived organic matrix and recrystallized during the drying process.

The particularly intense reflection observed near 2θ = 35.4° for AgCUR-EtOH NPs should not be interpreted automatically as evidence of a higher total AgNO_3_ concentration. Powder diffraction intensities can be affected by preferred crystallite orientation, particle packing, crystallite morphology, and sample preparation [59,60]. In addition, the two experimental profiles were normalized independently. Consequently, differences between individual normalized peak intensities provide information regarding the relative diffraction profiles but do not permit direct quantitative comparison of phase abundance between the formulations. The weaker reflections corresponding to the characteristic crystallographic planes of face-centered cubic Ag indicate that at least part of the silver precursor was reduced to crystalline metallic silver. However, the low intensity and partial overlap of these reflections with AgNO_3_-related peaks prevented a reliable quantitative assessment of the metallic phase. Therefore, the XRD results support metallic Ag formation but do not demonstrate complete conversion of Ag^+^ or the formation of a phase-pure metallic Ag product.

The Debye–Scherrer analysis yielded apparent coherent crystallite sizes of 82.0 ± 3.1 nm for the residual AgNO_3_ phase in AgCUR-EtOH NPs and 68.2 ± 5.6 nm in AgCUR-H_2_O NPs. These values characterize the coherent diffraction domains of the residual crystalline precursor. The larger apparent value obtained for the ethanolic formulation may indicate differences in AgNO_3_ recrystallization or crystal growth during drying; however, it does not demonstrate a higher phase abundance. Moreover, because instrumental broadening was not subtracted and only four comparatively resolved reflections were considered, the calculated values should be regarded as approximate apparent crystallite-size estimates rather than absolute crystallite dimensions [35,36,61,62].

Several additional weak reflections showed correspondence with the Ag_6_O_2_ reference pattern, suggesting a possible minor oxidized silver contribution. Such a phase might arise through partial oxidation during synthesis, drying, storage, or exposure of the silver-containing structures to air. Nevertheless, because the diagnostic reflections were weak and overlapped with those of AgNO_3_ and metallic Ag, this assignment remains tentative. Definitive differentiation of the silver oxidation states would require a complementary surface-sensitive technique, such as X-ray photoelectron spectroscopy. The detection of residual crystalline AgNO_3_ is also relevant to the interpretation of the biological findings. Unreacted or matrix-associated silver nitrate may act as a source of bioavailable Ag^+^ ions and could contribute to the concentration-dependent effects observed in HGF-1 cells. Accordingly, the cellular response cannot be attributed exclusively to metallic Ag nanoparticles. Instead, it may reflect the combined contribution of metallic and potentially oxidized silver-containing structures, residual ionic silver, and extract-derived phytochemicals associated with the particle surface. The relative contribution of these components may differ between the two formulations because the ethanolic and aqueous extracts represent chemically distinct reducing and stabilizing matrices. Differences in phytochemical composition may affect Ag^+^ reduction efficiency, nucleation, crystal growth, organic capping, and retention of the silver precursor. However, because crystalline phase identification was qualitative and quantitative phase fractions were not determined, solvent-dependent differences in silver conversion should be regarded as plausible interpretations rather than demonstrated quantitative mechanisms.

FTIR spectroscopy complemented the XRD findings by providing information regarding the organic functional groups and inorganic vibrational contributions present in the dried CUR-AgNP formulations. Both spectra exhibited broad O–H stretching bands around 3400 cm^−1^ and signals associated with aromatic, conjugated carbonyl, and C–O-containing groups. Such vibrations are characteristic of curcuminoids and other oxygen-containing phytochemicals, including polyphenols, carbohydrates, and related extract constituents [18,63,64,65]. Their presence indicates that an organic fraction derived from the *Curcuma longa* extracts remained associated with the final dried silver-containing formulations.

AgCUR-EtOH NPs exhibited a more clearly differentiated organic fingerprint, including the band near 2924 cm^−1^ assigned to aliphatic C–H stretching, the aromatic or conjugated vibrations around 1591 and 1510 cm^−1^, and the phenolic/enolic C–O-related contribution near 1271 cm^−1^. By comparison, several of these bands were less clearly resolved in AgCUR-H_2_O NPs. This difference is consistent with the previously reported chemical distinction between the CUR-EtOH and CUR-H_2_O extracts, which differed in extraction yield, phenolic content, antioxidant activity, and targeted phytochemical profile [20]. The FTIR findings therefore support solvent-dependent differences in the organic matrices retained within the final formulations.

The oxygen-containing functional groups detected by FTIR could participate in interactions with the silver-containing structures through adsorption, hydrogen bonding, electrostatic interactions, or coordination. Such groups may also contribute to the negative apparent zeta-potential values measured for the two formulations [18,65]. Nevertheless, FTIR spectra of the original extracts were not recorded under the same experimental conditions, and the analysis was performed on the final dried powders rather than on isolated nanoparticle surfaces. Consequently, the present results support the association of extract-derived organic constituents with the formulations but do not independently prove a specific capping arrangement or coordination mechanism. The most intense FTIR contribution in both formulations occurred around 1380 cm^−1^ and was assigned predominantly to the asymmetric stretching vibration of nitrate. The strong feature near 824 cm^−1^ was similarly compatible with an out-of-plane nitrate deformation mode. Additional nitrate-related vibrations may overlap with signals near approximately 1030–1040 cm^−1^ and with the feature observed around 1755–1761 cm^−1^ [66,67,68,69]. The FTIR results therefore corroborate the XRD identification of residual crystalline AgNO_3_ and provide independent spectroscopic evidence that nitrate-containing precursor-derived species remained in the dried formulations.

The prominent nitrate bands should not be interpreted quantitatively. FTIR intensities obtained using separately prepared KBr pellets may vary according to the sample-to-KBr ratio, pellet thickness, particle distribution, and pellet homogeneity. Accordingly, the relative depths of the nitrate-associated bands do not provide a reliable comparison of the amount of residual AgNO_3_ in AgCUR-EtOH NPs and AgCUR-H_2_O NPs. Quantitative determination would require a validated calibration procedure or an independent chemical assay. The weak signal detected near 523 cm^−1^ in AgCUR-H_2_O NPs may be compatible with an Ag–O lattice vibration. Ag–O-related FTIR signals have been reported in the approximately 500–535 cm^−1^ region for silver oxide-containing materials [70,71,72]. This observation is potentially consistent with the minor oxidized silver contribution tentatively suggested by XRD. However, the assignment cannot be considered definitive because the low-wavenumber region may contain overlapping lattice and organic fingerprint vibrations, and the oxidation state of silver was not independently confirmed.

Collectively, the FTIR findings indicate that the final CUR-AgNP powders comprised silver-containing crystalline structures associated with extract-derived organic constituents and residual nitrate-containing components. The more differentiated organic fingerprint of AgCUR-EtOH NPs may reflect the more chemically diverse phytochemical matrix obtained through ethanolic extraction, whereas the AgCUR-H_2_O NPs profile showed fewer resolved organic features. These compositional differences may contribute to the formulation-dependent hydrodynamic and biological behavior observed in the present study; however, a direct causal relationship cannot be established from FTIR analysis alone.

TEM and EDX provided complementary confirmation of Ag-containing nanoparticle formation. Both AgCUR-EtOH NPs and AgCUR-H_2_O NPs displayed polydisperse morphologies, with nanoscale particles coexisting with larger irregular structures or aggregates. The approximate particle-size ranges observed in this study, 15–175 nm for AgCUR-EtOH NPs and 15–150 nm for AgCUR-H_2_O NPs, were broader than those reported in several previous *Curcuma longa*-related AgNP studies. For example, Shameli et al. reported particles with a mean diameter of 6.30 ± 2.64 nm, Singh et al. described spherical AgNPs of approximately 25–30 nm, while Venkatadri et al. reported AgNPs in the 20–51 nm range [15,49,50]. The broader size range observed here may be explained by differences in extract composition, reaction conditions, and post-synthesis processing. In particular, the nanoparticles were recovered as dried powders, hand-ground, redispersed in distilled water, and sonicated before TEM analysis, which may favor partial aggregation or visualization of phytochemical matrix-associated clusters rather than exclusively isolated primary nanoparticles. The hydrodynamic dimensions determined by DLS were larger than the primary nanoscale structures visualized by TEM. This difference is expected because the two techniques assess distinct particle characteristics. TEM provides local information on dried structures deposited on a support grid, whereas DLS measures the translational diffusion of scattering entities dispersed in a liquid medium [73,74,75,76]. The polydisperse morphology observed by electron microscopy is consistent with the PDI values, which indicate broad and heterogeneous hydrodynamic size distributions.

The EDX spectra confirmed the presence of silver in both samples, supporting the formation of Ag-containing nanostructures. Similar electron microscopy/EDX-based approaches have been used in previous *Curcuma longa*-mediated AgNP studies to confirm silver formation [15,17]. In the present work, the intense C and O signals should be interpreted in relation to the organic phytochemical matrix, the carbon-coated copper grids, and the additional carbon coating used during sample preparation, rather than as evidence of sample contamination. Moreover, the Ag weight percentages obtained by EDX represent local values from the analyzed regions and should not be considered a global quantitative composition of the entire nanoparticle powder. Overall, the combined UV–Vis, DLS, zeta-potential, XRD, FTIR, TEM, and EDX results support the formation and physicochemical differentiation of the CUR-AgNP formulations, while also demonstrating that the final dried materials contained heterogeneous silver-associated structures, extract-derived organic constituents, and residual precursor-related components. Furthermore, the DLS results should not be directly extrapolated to the biological exposure conditions. The cell-culture stock dispersions were subsequently prepared in ultrapure distilled water and diluted in culture medium, whereas the AgCUR-EtOH NPs physicochemical measurement was performed in ethanol. Protein adsorption, ionic strength, and interactions with cell-culture medium components may substantially alter particle size and surface behavior. Therefore, the relationship between the current DLS findings and the different cellular responses of the two formulations remains indirect and should not be interpreted as a demonstrated causal association. Overall, the use of complementary DLS, zeta-potential, XRD, FTIR, and electron-microscopy analyses is consistent with previous physicochemical characterization studies of *Curcuma longa*-mediated AgNP systems [77,78].

The present antibacterial evaluation demonstrated a clear strain-dependent response to the two CUR-AgNP formulations. *S. mutans* was the most susceptible microorganism, with MIC values below 10 µg/mL for both AgCUR-EtOH NPs and AgCUR-H_2_O NPs. By contrast, substantially higher concentrations were required to inhibit *S. aureus* and particularly *S. oralis*. AgCUR-H_2_O NPs consistently exhibited lower MIC and MBC values than AgCUR-EtOH NPs against all three tested strains, indicating a comparatively stronger antibacterial effect under the applied experimental conditions. These findings are particularly relevant when considered in relation to the antimicrobial behavior of the original CUR-EtOH and CUR-H_2_O extracts from which the nanoparticle formulations were synthesized. In the previous study, CUR-EtOH exhibited stronger antibacterial activity against the oral streptococci than CUR-H_2_O. The ethanolic extract showed a MIC of 10 µL/well against *S. mutans* and 100 µL/well against *S. oralis*, whereas CUR-H_2_O showed a MIC of 60 µL/well against *S. mutans* and no detectable MIC against *S. oralis* within the investigated range [20]. After silver nanoparticle synthesis, however, the relative antibacterial pattern was reversed: AgCUR-H_2_O NPs showed lower MIC and MBC values than AgCUR-EtOH NPs against both streptococcal strains and against *S. aureus*. This reversal suggests that the antibacterial activity of the final formulations was no longer governed exclusively by the antimicrobial properties of the parent phytochemical extracts. Instead, the biological effect likely reflected the combined influence of silver-containing structures, silver-species availability, particle dispersion, surface-associated phytochemicals, residual precursor-related components, and strain-specific susceptibility.

Direct numerical comparison between the antibacterial activity of the original extracts and that of the CUR-AgNP formulations is not possible because the extract study initially expressed MIC values as stock-extract volumes per well and corresponding crude-extract mass per well, whereas the present results are expressed as µg/mL of total dried nanoparticle formulation. The starting extract concentrations were approximately 98 mg/mL, and the tested volumes corresponded to milligram quantities of crude extract per well. Consequently, the most appropriate comparison concerns the relative susceptibility patterns rather than a direct calculation of fold enhancement following nanoparticle synthesis.

The greater activity of AgCUR-H_2_O NPs may be related, at least partly, to the physicochemical characteristics identified in the present study. AgCUR-H_2_O NPs showed a substantially lower Z-average hydrodynamic diameter than AgCUR-EtOH NPs and displayed a more distinguishable population of small nanoscale structures by TEM. Smaller or more effectively dispersed silver-containing structures may provide a larger accessible surface area and facilitate interaction with bacterial envelopes and release of bioavailable silver species. Nevertheless, this interpretation remains tentative because the DLS measurements were performed in different dispersants and because neither the amount of elemental silver nor the silver-ion-release profile was quantified.

The XRD and FTIR findings must also be considered when interpreting the antimicrobial data. Both dried formulations contained residual crystalline AgNO_3_ and prominent nitrate-associated FTIR bands. Because the powders were recovered without an additional washing step, the measured antibacterial activity cannot be attributed exclusively to metallic AgNPs. Instead, the observed MIC and MBC values may reflect the combined effects of metallic and potentially oxidized silver-containing structures, silver species released from the nanoparticles, retained precursor-derived silver, and extract-associated phytochemicals. The absence of an AgNO_3_-only control and of corresponding CUR-EtOH and CUR-H_2_O extract controls within the same antimicrobial experiment prevents experimental separation of these individual contributions.

The strong effect against *S. mutans* is broadly consistent with previous findings for Curcuma-mediated silver nanoparticles. Thomas et al. reported a MIC of 7.8 µg/mL and an MBC of 125 µg/mL for *Curcuma aromatica*-mediated AgNPs against *S. mutans*. The MIC values obtained in the present study, approximately 7 and 9 µg/mL, were therefore within a comparable range, whereas the MBC values of 62 and 88 µg/mL were lower. Nevertheless, the comparison should remain cautious because the previous formulation was produced from a different *Curcuma* species and using different synthesis, purification, and microbiological procedures [79]. For *S. aureus*, Amin et al. reported MIC and MBC values of 150 and 200 µg/mL, respectively, for AgNPs synthesized using an aqueous *Curcuma longa* rhizome extract. The values obtained for AgCUR-H_2_O NPs in the present study (138 and 192 µg/mL), were broadly comparable, whereas AgCUR-EtOH NPs showed slightly higher MIC and MBC values of 168 and 266 µg/mL [78]. By comparison, Sarău et al. reported MIC and MBC values of 97 and 164 µg/mL against *S. aureus* for *Punica granatum*-derived AgNPs synthesized using a similar high-concentration AgNO_3_ protocol [33]. These differences emphasize that the use of a similar silver precursor concentration does not necessarily produce identical antimicrobial activity, because the extract composition, nanoparticle morphology, surface chemistry, aggregation state, purification procedure, and availability of silver species may differ substantially among formulations. A broader study of non-plant-specific AgNPs reported identical MIC and MBC values of 625 µg/mL against *S. aureus*, considerably higher than the values obtained for both CUR-AgNP formulations. However, such comparisons are influenced by particle characteristics, concentration-expression methods, and susceptibility-testing procedures and should not be regarded as evidence of intrinsic superiority without standardized side-by-side testing [80].

The relationship between MIC and MBC further illustrates the strain-dependent nature of the response. Based on the experimental values, the MBC/MIC ratios were approximately 9.8 and 8.9 against *S. mutans* for AgCUR-EtOH NPs and AgCUR-H_2_O NPs, respectively. In contrast, the corresponding ratios were approximately 1.6 and 1.4 against *S. aureus* and 2.0 and 2.4 against *S. oralis*. Under the conventional in vitro criterion, an MBC/MIC ratio of ≤4 is considered compatible with a predominantly bactericidal effect, whereas a ratio > 4 indicates a predominantly bacteriostatic effect [81,82]. Therefore, both CUR-AgNP formulations displayed a bactericidal-type profile against *S. aureus* and *S. oralis*, while their initial activity against *S. mutans* was predominantly growth-inhibitory, with higher concentrations required to achieve bactericidal activity. This classification should be interpreted cautiously because it is an operational in vitro definition and may depend on the strain, experimental methodology, and concentration intervals used.

The antimicrobial findings should also be interpreted together with the HGF-1 cytocompatibility results. AgCUR-H_2_O NPs showed both the stronger antibacterial effect and the more pronounced reduction in HGF-1 viability. At 10 µg/mL, HGF-1 viability was 52.14% following exposure to AgCUR-H_2_O NPs and 71.88% following exposure to AgCUR-EtOH NPs. This parallel trend may indicate greater overall biological reactivity or higher bioavailability of antibacterial silver species in the aqueous-extract-mediated formulation, rather than selective toxicity toward bacteria. Importantly, the MIC values against *S. mutans* fell within the 1–10 µg/mL concentration range evaluated on HGF-1 cells. AgCUR-EtOH NPs therefore combined an MIC of approximately 9 µg/mL with a higher measured HGF-1 viability at 10 µg/mL than AgCUR-H_2_O NPs under the applied experimental conditions. In contrast, the MIC values required against *S. aureus* and *S. oralis* were considerably higher than the maximum concentration evaluated on HGF-1 cells. Consequently, the cytocompatibility results obtained up to 10 µg/mL cannot be extrapolated to the substantially higher antibacterial concentrations required for these two strains.

The present findings concern exclusively planktonic bacterial growth. No biofilm formation or preformed-biofilm assay was performed; therefore, the MIC and MBC results must not be interpreted as evidence of antibiofilm activity. Further studies should determine the minimum biofilm inhibitory concentration, evaluate the biomass and viability of established mono- and multispecies oral biofilms, and distinguish the contribution of metallic AgNPs from that of residual precursor-derived silver through comparison with washed nanoparticle formulations, parent extracts, and an AgNO_3_ control.

The next step was to evaluate the cytocompatibility profile of AgCUR-EtOH NPs and AgCUR-H_2_O NPs using HGF-1 human gingival fibroblasts as an in vitro oral-relevant cell model. Gingival fibroblasts represent a major cellular component of the gingival connective tissue and are directly involved in extracellular matrix homeostasis, wound healing, and inflammatory responses. Therefore, the use of HGF-1 cells is appropriate for a preliminary screening of nanomaterials intended for potential dental or oral-health-oriented applications. Similar HGF-1-based models have been used to assess the cytocompatibility of dental materials, plant-derived compounds, and AgNP-based systems [21,24,83]. The concentration range investigated in HGF-1 cells allowed the characterization of formulation-dependent effects at comparatively low exposure levels and the identification of differences in cytocompatibility between AgCUR-EtOH NPs and AgCUR-H_2_O NPs.

The biological evaluation started with the MTT assay, which estimates cell viability based on the ability of metabolically active cells to reduce MTT to formazan crystals. Although MTT is widely used as a first-line cytotoxicity screening assay, it mainly reflects mitochondrial/metabolic activity and should not be interpreted as a stand-alone confirmation of cell death [22,84,85]. In the present study, both CUR-AgNP formulations induced a concentration-dependent reduction in HGF-1 viability after 24 h of exposure. AgCUR-EtOH NPs maintained viability close to the control at 1–3 µg/mL and above 80% up to 5 µg/mL, whereas viability decreased to 71.88% at 10 µg/mL. In contrast, AgCUR-H_2_O NPs produced a stronger reduction in viability, reaching 52.14% at 10 µg/mL. These results indicate that AgCUR-EtOH NPs had a more favorable metabolic cytocompatibility profile, while AgCUR-H_2_O NPs exerted a more pronounced concentration-dependent effect on HGF-1 cells. These findings are consistent with previous reports showing that AgNP-induced cytotoxicity in oral fibroblast models is highly dependent on concentration, particle size, surface functionalization, and stabilizing/capping agents. Niska et al. demonstrated that 10 nm AgNPs reduced HGF-1 viability after 24 h and that the capping agent strongly influenced both cytotoxicity and antimicrobial activity; AgNPs capped with lipoic acid or polyethylene glycol were less cytotoxic than uncapped or tannic-acid-capped AgNPs [21]. Halkai et al. also reported concentration-dependent cytotoxicity of fungal-derived AgNPs on human gingival fibroblasts using the MTT assay [86]. In primary human periodontal fibroblasts, Hernández-Sierra et al. showed that smaller AgNPs, particularly particles below 20 nm, produced more evident dose- and time-dependent cytotoxicity, whereas larger 80–100 nm particles did not markedly affect cell viability under their tested conditions [87]. Together, these studies support the interpretation that the biological response to AgNPs cannot be attributed only to the presence of silver but depends on the physicochemical and surface properties of the nanoparticle system. Although different nanoparticles and experimental conditions were used, a similar concentration-dependent decrease in cell viability has been reported in the literature. For instance, Rao Setvaji et al. evaluated the cytotoxicity of strontium fluorapatite nanoparticles (SrFAp NPs) from *Equisetum arvense* plant extract on human gingival fibroblasts, reporting a reduction in viability from 83% at 10 μg/mL to 70% at 80 μg/mL [88]. Likewise, Lelapityamit et al. demonstrated that gold nanoparticles exert concentration-dependent cytotoxic effects on HGF cells, with minimal impact at low doses and more pronounced effects at higher concentrations [89].

In the present study, bright-field microscopy further supported the MTT findings. AgCUR-EtOH NPs caused only a slight reduction in confluence, without major alterations in the typical fibroblast-like morphology across most of the tested concentration range. Conversely, AgCUR-H_2_O NPs induced more evident morphological changes, including reduced confluence, cell rounding, and shrinkage, especially at 5 and 10 µg/mL. Such morphological changes are commonly associated with cellular stress and reduced adhesion or viability, and have also been described in previous studies investigating AgNPs or other nanoparticle-containing dental materials on oral cell models [21,90].

To complement the MTT results, the NRU assay was used to assess lysosomal integrity. This was important because cell viability is a multidimensional parameter, and metabolic assays alone may not fully capture early cellular stress or organelle-specific impairment. Neutral red is taken up by viable cells and accumulates in lysosomes; therefore, reduced neutral red uptake may reflect impaired lysosomal function and/or decreased cell viability [91]. In the present study, AgCUR-EtOH NPs preserved lysosomal neutral red uptake better than AgCUR-H_2_O NPs, with NRU values remaining near the control at 1–4 µg/mL and decreasing to 79.87% at 10 µg/mL. By contrast, AgCUR-H_2_O NPs caused a clearer concentration-dependent reduction in NRU, reaching 61.99% at 10 µg/mL. This pattern confirms that the aqueous-extract-mediated nanoparticles exerted a stronger impact not only on mitochondrial metabolic activity, but also on lysosomal function. Similar NRU-based approaches have been applied for the cytocompatibility assessment of plant-derived preparations on human gingival fibroblasts using *Mimusops elengi* extract [91].

Mitochondrial function was further investigated using JC-1 staining, because mitochondrial membrane potential is an early and sensitive indicator of cellular stress and pro-apoptotic signaling. JC-1 accumulates in polarized mitochondria as red-emitting aggregates, whereas mitochondrial depolarization shifts the signal toward green-emitting monomers; therefore, the aggregate/monomer fluorescence ratio reflects mitochondrial membrane potential [92]. In the present study, AgCUR-EtOH NPs produced a moderate decrease in the JC-1 aggregate/monomer ratio, with the value remaining at 74.41% at 10 µg/mL. In contrast, AgCUR-H_2_O NPs caused a more pronounced mitochondrial response, with the ratio decreasing to 48.33% at 10 µg/mL. These findings indicate that AgCUR-H_2_O NPs induced stronger mitochondrial depolarization, consistent with the MTT and NRU results. This mitochondrial response is biologically relevant because mitochondrial dysfunction has been increasingly recognized as an important contributor to oral inflammatory and periodontal pathologies. Mitochondria regulate energy metabolism, reactive oxygen species generation, calcium homeostasis, and apoptosis-related signaling, and their functional status is therefore important for maintaining periodontal and gingival tissue homeostasis [93,94]. AgNPs are known to induce cellular stress through mechanisms involving oxidative stress, mitochondrial dysfunction, membrane damage, and apoptosis-related pathways, although the magnitude of these effects varies depending on particle size, surface chemistry, dose, and cell type [95,96]. Thus, the stronger mitochondrial response observed for AgCUR-H_2_O NPs may partly explain their lower viability and NRU values compared with AgCUR-EtOH NPs.

The additional MitoTracker^TM^ Red CMXRos/Hoechst 33342 staining provided complementary information on mitochondrial organization and nuclear morphology. AgCUR-EtOH NPs largely preserved mitochondrial and nuclear morphology, with only mild changes at the highest concentration. The apoptotic index increased from 3.93% to 10.10% across the tested concentration range, suggesting a mild concentration-associated cellular stress response. In contrast, AgCUR-H_2_O NPs induced more evident mitochondrial and nuclear alterations, particularly from 4 µg/mL onward, and the apoptotic index increased from 5.22% to 13.60%. These findings are in agreement with the JC-1 results and suggest that mitochondrial depolarization was accompanied by mild nuclear alterations, especially in the AgCUR-H_2_O NP-treated cells. However, because apoptosis was assessed by fluorescence morphology rather than by molecular markers such as caspase activation, annexin V binding, or DNA fragmentation assays, these changes should be interpreted as apoptotic-like features rather than definitive proof of apoptosis induction.

AO/PI staining further supported this interpretation. Across the tested concentrations, both formulations showed a predominance of green-stained cells, indicating that most cells maintained membrane integrity after 24 h of exposure. AgCUR-EtOH NPs induced only mild apoptotic-like nuclear changes, mainly at the highest concentration, while sporadic PI-positive cells remained comparable to the untreated control. AgCUR-H_2_O NPs produced more visible apoptotic-like features and a gradual reduction in cell confluence, particularly at 5 and 10 µg/mL, but without a marked increase in PI-positive cells. Because PI enters cells with compromised plasma membrane integrity, these findings suggest that the tested CUR-AgNPs induced mainly mild stress/apoptotic-like changes rather than extensive necrotic membrane damage under the tested conditions [97,98].

Overall, the in vitro results show a coherent biological pattern across complementary endpoints. AgCUR-EtOH NPs displayed the more favorable cytocompatibility profile in HGF-1 cells, particularly at concentrations up to 5 µg/mL, with preserved morphology, limited lysosomal impairment, moderate mitochondrial effects, and only mild apoptotic-like changes. AgCUR-H_2_O NPs showed a stronger concentration-dependent impact, reflected by lower MTT viability, reduced NRU, more pronounced mitochondrial depolarization, and higher apoptotic index values. These differences may be related to the distinct phytochemical matrices of the two extracts, which can influence nanoparticle formation, surface chemistry, aggregation state, silver availability, and cellular interactions. In this context, the previously reported solvent-dependent differences between CUR-EtOH and CUR-H_2_O extracts are relevant because the phytochemical composition of the reducing/capping matrix may modulate not only nanoparticle synthesis and stabilization but also the biological behavior of the final AgNP formulation [20].

Importantly, these findings should be interpreted as a preliminary cytocompatibility screening rather than as a complete safety assessment. The study was performed after 24 h of exposure using a 2D monoculture model, and the tested concentration range was limited to 1–10 µg/mL. Future studies should include longer exposure times, additional oral cell types, 3D oral mucosal or periodontal models, oxidative stress markers, inflammatory mediators, silver ion release, and more detailed apoptosis/necrosis assays. These additional endpoints would help clarify whether the observed differences between AgCUR-EtOH NPs and AgCUR-H_2_O NPs are driven mainly by nanoparticle size/aggregation, surface phytochemical coating, Ag release, or differential cellular uptake.

The HET-CAM assay was included as an additional screening step to evaluate the acute irritant potential of AgCUR-EtOH NPs and AgCUR-H_2_O NPs at the level of a highly vascularized biological membrane. This model is widely used as an alternative approach for assessing mucosal and ocular irritation potential because it allows direct visualization of early vascular events such as hemorrhage, vascular lysis, and coagulation after topical application of a test substance [26,27,99,100]. In nanotoxicology, the CAM model is also considered useful for preliminary vascular compatibility screening, since it provides rapid information on how nanomaterials interact with a living vascular network under short-term exposure conditions [28].

In the present study, distilled water was used as the negative control and showed an irritation score of 0.07, confirming the absence of relevant vascular irritation. Conversely, 1% SDS produced a high irritation score of 19.73, associated with hemorrhage, vascular lysis, and coagulation, thereby confirming the sensitivity and responsiveness of the experimental model. According to commonly used HET-CAM irritation-score classifications, scores below 0.9 are generally considered non-irritant, scores between 1 and 4.9 indicate slight or weak irritation, scores between 5 and 8.9 indicate moderate irritation, and scores above 9 indicate strong irritation [26,45]. Based on this grading system, AgCUR-H_2_O NPs, with an IS value of 0.69, were classified as non-irritant, whereas AgCUR-EtOH NPs, with an IS value of 1.06, were classified as slight or weak irritant, at the lowest end of this category. However, this value remained close to the non-irritant threshold and markedly lower than that of the positive control, indicating only a very low acute irritant response.

The stereomicroscopic evaluation further supported this interpretation. After 5 min of exposure, neither AgCUR-H_2_O NPs nor AgCUR-EtOH NPs produced marked vascular damage comparable to the SDS-treated CAM. The vascular plexus remained largely preserved, and the recorded events occurred late during the observation period, particularly for vascular lysis and coagulation. This is important because, in the HET-CAM scoring system, early-onset vascular reactions contribute more strongly to the final irritation score than late or absent reactions. Therefore, the low IS values obtained for both CUR-AgNP formulations indicate limited acute vascular irritation after direct CAM exposure.

These findings are consistent with previous reports suggesting that AgNPs may show acceptable HET-CAM compatibility depending on their concentration, size, formulation, and surface chemistry. Freire et al. evaluated AgNP colloids using the hen’s egg test and reported that the tested AgNPs did not promote vasoconstriction, hemorrhage, or coagulation, while also inhibiting *Streptococcus mutans* biofilm formation on dental enamel [29]. This is particularly relevant for the present study because it links AgNP-based systems with both oral-health-oriented antimicrobial applications and CAM-based irritation screening. However, other studies have shown that biogenic AgNPs can also modulate CAM vascular behavior, especially when evaluated for antiangiogenic effects. For example, Baharara et al. reported antiangiogenic effects of biogenic AgNPs synthesized using *Salvia officinalis* extract on the chick CAM model [101]. These data emphasize that CAM responses to AgNPs depend strongly on the nanoparticle system, the biological coating, the concentration, and the endpoint evaluated.

The in ovo results should also be interpreted together with the in vitro cytocompatibility data. Although AgCUR-H_2_O NPs induced a stronger concentration-dependent response in HGF-1 cells compared with AgCUR-EtOH NPs, their HET-CAM irritation score was slightly lower than that of AgCUR-EtOH NPs. This apparent difference should not be considered contradictory, because the two models evaluate different biological endpoints. The in vitro assays assessed metabolic activity, lysosomal integrity, mitochondrial membrane potential, nuclear morphology, and membrane integrity in gingival fibroblasts after 24 h of exposure. In contrast, the HET-CAM assay evaluated acute vascular irritation after only 5 min of direct exposure to the CAM. Therefore, the HET-CAM results primarily support the absence of major acute vascular irritation under the tested conditions, but they do not replace longer-term cytocompatibility, inflammatory, angiogenic, or systemic safety assessments. Moreover, the response of the standalone CUR-AgNP suspensions cannot be directly extrapolated to their behavior after incorporation into a cured dental or orthodontic adhesive matrix.

Overall, the HET-CAM assay supports a favorable preliminary in ovo biocompatibility profile for both CUR-AgNP formulations at 10 µg/mL. AgCUR-H_2_O NPs can be classified as non-irritant under the tested conditions, whereas AgCUR-EtOH NPs showed only a very low/slight irritant response, close to the non-irritant threshold. Together with the in vitro findings, these results suggest that AgCUR-EtOH NPs produced less pronounced effects on the evaluated HGF-1 cellular endpoints under the tested conditions, while both formulations show low acute vascular irritation in the CAM model. Nevertheless, further studies are required to evaluate longer exposure times, repeated-dose effects, inflammatory mediators, angiogenic markers, and additional oral-relevant models before definitive conclusions regarding clinical or dental safety can be drawn. The present findings provide the physicochemical and biological basis for selecting and optimizing CUR-AgNP formulations for subsequent incorporation into experimental dental or orthodontic adhesive systems.

## 5. Study Limitations and Future Directions

The present study was designed as a comparative evaluation of the biosynthesis, physicochemical characteristics, antibacterial activity, and preliminary biological compatibility of AgCUR-EtOH NPs and AgCUR-H_2_O NPs as standalone nanoformulations. The formulations were compared at equivalent concentrations expressed as total dried powder mass. However, the total silver content, extract-derived organic fraction, residual precursor content, and silver-species-release profiles were not quantitatively determined. Consequently, the administered doses cannot be considered compositionally equivalent, and the observed differences should be interpreted as formulation-dependent responses under the tested experimental conditions rather than as definitive evidence of the intrinsic superiority of one nanoparticle system. Quantitative compositional characterization and silver-release studies in biologically relevant media will therefore be included in the subsequent development stage.

The antibacterial evaluation was limited to MIC and MBC determination against planktonic cultures of three Gram-positive bacterial strains and did not include biofilm models. Moreover, the cytocompatibility and HET-CAM assessments represented preliminary short-term screening endpoints and cannot be directly extrapolated to the behavior of the nanoformulations after incorporation into a polymeric dental or orthodontic material. Future studies will focus on incorporating AgCUR-EtOH NPs and AgCUR-H_2_O NPs into experimental adhesive systems and identifying a nanoparticle loading that preserves antibacterial activity while maintaining cytocompatibility and material performance. Thus, future investigations will determine whether the properties observed for the standalone nanoformulations can be retained within a clinically relevant adhesive platform.

## 6. Conclusions

This study demonstrated the feasibility of using turmeric powder-derived *Curcuma longa* ethanolic and aqueous extracts as distinct phytochemical matrices for the green synthesis of silver-containing nanoformulations. The complementary physicochemical analyses revealed formulation-dependent characteristics. UV–Vis spectroscopy showed matrix-dependent optical profiles and supported the progression of the synthesis reactions, whereas DLS demonstrated broad and polydisperse hydrodynamic distributions after redispersion. Under their respective solvent-specific measurement conditions, AgCUR-H_2_O NPs exhibited a lower Z-average hydrodynamic diameter than AgCUR-EtOH NPs, while both formulations displayed negative apparent zeta-potential values. XRD indicated heterogeneous crystalline compositions dominated by residual crystalline AgNO_3_, together with weaker reflections consistent with metallic Ag and a possible minor oxidized silver contribution. FTIR analysis identified extract-derived oxygen-containing functional groups and prominent nitrate-associated bands, supporting the coexistence of phytochemical and precursor-derived components in the final dried formulations. TEM and EDX further confirmed the presence of Ag-containing nanostructures with polydisperse morphologies and approximate particle-size ranges of 15–175 nm for AgCUR-EtOH NPs and 15–150 nm for AgCUR-H_2_O NPs. Taken together, these findings show that the obtained powders represent complex silver-containing nanoformulations rather than compositionally uniform populations of purified metallic AgNPs.

Both formulations exhibited antibacterial activity against the three tested Gram-positive strains, with *S. mutans* showing the highest susceptibility. At equivalent concentrations expressed as total dried formulation mass, AgCUR-H_2_O NPs produced consistently lower MIC and MBC values than AgCUR-EtOH NPs against *S. mutans*, *S. aureus*, and *S. oralis*. For *S. mutans*, MIC values were 7 and 9 µg/mL and MBC values were 62 and 88 µg/mL for AgCUR-H_2_O NPs and AgCUR-EtOH NPs, respectively. These results demonstrate relevant activity against planktonic oral bacteria but should not be extrapolated to biofilm inhibition or eradication.

The HGF-1 assays showed different formulation-dependent cellular responses under the tested conditions. At equivalent total formulation concentrations, AgCUR-EtOH NPs produced a less pronounced reduction in cell viability, lysosomal function, mitochondrial membrane potential, and cellular and nuclear integrity than AgCUR-H_2_O NPs. At 10 µg/mL, HGF-1 viability was 71.88% for AgCUR-EtOH NPs and 52.14% for AgCUR-H_2_O NPs. Nevertheless, these differences cannot be attributed exclusively to nanoparticle size, extraction solvent, or intrinsic silver nanoparticle properties because the actual silver, organic, and residual precursor contents of the two powders were not quantitatively normalized.

Both formulations showed low acute vascular irritation in the HET-CAM model at 10 µg/mL. AgCUR-H_2_O NPs were classified as non-irritant, whereas AgCUR-EtOH NPs produced only a very low/slight irritation response close to the non-irritant threshold. The difference between the HGF-1 and HET-CAM responses reflects the distinct endpoints and exposure periods assessed by the two preliminary biological models and does not represent contradictory evidence.

Overall, the present study provides a physicochemical, antibacterial, and preliminary biological basis for the further development of CUR-AgNP-containing oral biomaterials. AgCUR-EtOH NPs and AgCUR-H_2_O NPs should be regarded as compositionally distinct formulations that produced different antibacterial and cellular responses under the applied experimental conditions, rather than as biologically equivalent doses of purified AgNPs. Future work will focus on quantitative compositional characterization, silver-species-release assessment, evaluation in oral biofilm models, and incorporation of the nanoformulations into experimental dental or orthodontic adhesive systems. Optimization of nanoparticle loading will be required to preserve antibacterial performance while maintaining cytocompatibility, polymerization behavior, adhesive properties, and the functional stability of the resulting material.

## Figures and Tables

**Table 1:** Hydrodynamic size, polydispersity index, and apparent zeta potential of the redispersed CUR-AgNP formulations.

Formulation	Dispersant	Z-Average Hydrodynamic Diameter (nm)	PDI	Apparent Zeta Potential (mV)
AgCUR-H_2_O NPs	Distilled water	264	0.416	−21.8 ± 0.8
AgCUR-EtOH NPs	Ethanol	1742	0.500	−20.4 ± 0.6

Zeta-potential values are expressed as mean ± standard deviation from five consecutive determinations. DLS and zeta-potential measurements were performed at 30 °C using the dispersant corresponding to each formulation.

**Table 2:** Main FTIR absorption bands observed for the dried CUR-AgNP formulations and their tentative vibrational assignments.

AgCUR-EtOH NPs (cm^−1^)	AgCUR-H_2_O NPs (cm^−1^)	Tentative Vibrational Assignment
~3400	~3400	O–H stretching of hydrogen-bonded phenolic/alcoholic groups and adsorbed moisture
2924	Not clearly resolved	Aliphatic C–H stretching
1755–1761	~1760	C=O stretching of carbonyl-containing constituents and/or nitrate combination bands
1591	1593	Aromatic C=C stretching and/or conjugated C=O vibrations
~1510	Not clearly resolved	Aromatic skeletal/ring vibration
1383	1377–1379	Predominantly asymmetric stretching of NO_3_^−^; possible overlap with organic deformation modes
1271	Not clearly resolved	Phenolic/enolic C–O stretching; possible nitrate-related overlap
1165	Weak/not clearly resolved	C–O and C–O–C stretching of oxygen-containing organic constituents
1032	~1038, weak	C–O/C–O–C stretching and/or nitrate symmetric stretching
~824	~824	Predominantly nitrate out-of-plane deformation; possible overlap with aromatic C–H deformation
Not clearly resolved	~523	Possible Ag–O lattice vibration; tentative assignment

Band assignments are tentative and may include overlapping vibrational contributions from extract-derived organic constituents and residual nitrate-containing species. Absolute transmittance intensities were not used for quantitative comparison because the spectra were obtained from separately prepared KBr pellets.

**Table 3:** Minimum inhibitory concentration and minimum bactericidal concentration of AgCUR-EtOH NPs and AgCUR-H_2_O NPs against the tested Gram-positive bacterial strains.

Bacterial Strain	AgCUR-EtOH NPs MIC (µg/mL)	AgCUR-EtOH NPs MBC (µg/mL)	AgCUR-H_2_O NPs MIC (µg/mL)	AgCUR-H_2_O NPs MBC (µg/mL)
*Streptococcus mutans* ATCC 25175	9	88	7	62
*Staphylococcus aureus* ATCC 25923	168	266	138	192
*Streptococcus oralis* ATCC 9811	235	462	157	385

MIC, minimum inhibitory concentration; MBC, minimum bactericidal concentration. Values are expressed as µg/mL of total dried CUR-AgNP formulation in the final assay medium. The reported concentrations do not represent elemental silver or purified metallic silver nanoparticle concentrations.

**Table 4:** Irritation score (IS) and occurrence times of hemorrhage (t_H_), vascular lysis (t_L_), and coagulation (t_C_), recorded during the HET-CAM assay.

	H_2_O	1% SDS	AgCUR-H_2_O NPs	AgCUR-EtOH NPs
IS	0.07	19.73	0.69	1.06
t_H_ (s)	300	27	300	300
t_L_ (s)	300	20	290	283
t_C_ (s)	300	14	287	280

IS classification: 0.0–0.9, non-irritant; 1.0–4.9, slight or weak irritant; 5.0–8.9, moderately irritant; and 9.0–21.0, strongly or severely irritant.

## Data Availability

All the data obtained are included in the present article. Further inquiries can be directed to the corresponding authors.

## Abbreviations

The following abbreviations are used in this manuscript:

AgNPs	Silver Nanoparticles
CUR	*Curcuma longa*
CUR-EtOH	Turmeric powder-derived *Curcuma longa* ethanolic extract
CUR-H_2_O	Turmeric powder-derived *Curcuma longa* aqueous extract
CUR-AgNPs	*Curcuma longa* extract-mediated silver nanoparticles
AgCUR-EtOH NPs	Silver nanoparticles synthesized using CUR-EtOH extract
AgCUR-H_2_O NPs	Silver nanoparticles synthesized using CUR-H_2_O extract
AgNO_3_	Silver nitrate
UV-Vis	Ultraviolet–visible spectroscopy
TEM	Transmission electron microscopy
STEM	Scanning transmission electron microscopy
EDX	Energy-dispersive X-ray spectroscopy
HGF-1	Human gingival fibroblast cell line
DMEM	Dulbecco’s Modified Eagle’s Medium
FBS	Fetal bovine serum
PBS	Phosphate-buffered saline
DMSO	Dimethyl sulfoxide
MTT	3-(4,5-dimethylthiazol-2-yl)-2,5-diphenyltetrazolium bromide
NRU	Neutral red uptake
ΔΨm	Mitochondrial membrane potential
JC-1	5,5′,6,6′-tetrachloro-1,1′,3,3′-tetraethylbenzimidazolylcarbocyanine iodide
AO/PI	Acridine orange/propidium iodide
CAM	Chorioallantoic membrane
HET-CAM	Hen’s egg test–chorioallantoic membrane
SDS	Sodium dodecyl sulfate
IS	Irritation score
t_H_	Time of hemorrhage occurrence
t_L_	Time of vascular lysis occurrence
t_C_	Time of coagulation occurrence
SD	Standard deviation
ANOVA	Analysis of variance
DLS	Dynamic light scattering
PDI	Polydispersity index
XRD	X-ray diffraction
FWHM	Full width at half maximum
FTIR	Fourier-transform infrared spectroscopy
MIC	Minimum inhibitory concentration
MBC	Minimum bactericidal concentration
